# Nonlinear decoding of a complex movie from the mammalian retina

**DOI:** 10.1371/journal.pcbi.1006057

**Published:** 2018-05-10

**Authors:** Vicente Botella-Soler, Stéphane Deny, Georg Martius, Olivier Marre, Gašper Tkačik

**Affiliations:** 1 Institute of Science and Technology Austria, Klosterneuburg, Austria; 2 Sorbonne Université, INSERM, CNRS, Institut de la Vision, 17 rue Moreau, F-75012 Paris, France; 3 Max Planck Institute for Intelligent Systems, Tübingen, Germany; Imperial College London, UNITED KINGDOM

## Abstract

Retina is a paradigmatic system for studying sensory encoding: the transformation of light into spiking activity of ganglion cells. The inverse problem, where stimulus is reconstructed from spikes, has received less attention, especially for complex stimuli that should be reconstructed “pixel-by-pixel”. We recorded around a hundred neurons from a dense patch in a rat retina and decoded movies of multiple small randomly-moving discs. We constructed nonlinear (kernelized and neural network) decoders that improved significantly over linear results. An important contribution to this was the ability of nonlinear decoders to reliably separate between neural responses driven by locally fluctuating light signals, and responses at locally constant light driven by spontaneous-like activity. This improvement crucially depended on the precise, non-Poisson temporal structure of individual spike trains, which originated in the spike-history dependence of neural responses. We propose a general principle by which downstream circuitry could discriminate between spontaneous and stimulus-driven activity based solely on higher-order statistical structure in the incoming spike trains.

## Author summary

Neurons in the retina transform patterns of incoming light into sequences of neural spikes. We recorded from ∼100 neurons in the rat retina while it was stimulated with a complex movie. Using machine learning regression methods, we fit decoders to reconstruct the movie shown from the retinal output. We demonstrated that retinal code can only be read out with a low error if decoders make use of correlations between successive spikes emitted by individual neurons. These correlations can be used to ignore spontaneous spiking that would, otherwise, cause even the best linear decoders to “hallucinate” nonexistent stimuli. This work represents the first high resolution single-trial full movie reconstruction and suggests a new paradigm for separating spontaneous from stimulus-driven neural activity.

## Introduction

Decoding plays a central role in our efforts to understand the neural code [1–4]. While statistical analyses of neural responses can be used to directly estimate [5, 6] or bound [7] the information content of spike trains, such analyses remain agnostic about what the encoded bits might mean or how they could be read out [8]. In contrast, decoding provides an explicit computational procedure for recovering the stimulus from recorded single-trial neural responses, allowing us to ask not only “how much”, but also “what” the neural system encodes [9]. This is particularly relevant when a rich stimulus is represented by a large neural population—a regime which is increasingly accessible due to recent experimental progress, and the regime that we explore here.

Decoding from large populations presents a significant technical challenge due to its intrinsic high dimensionality. Past work has predominantly addressed this problem using two approaches. In the first approach, one only presents stimuli that have simple, low-dimensional representations, in order to turn decoding into a tractable fitting (e.g., angular velocity of a moving pattern [10], luminance flicker [11], 1D bar position [12], etc.) or classification problem (e.g., shape identity [13], a small set of orientations or velocities [14], etc.). It is unclear, however, how results for simple stimuli can be generalized to naturalistic stimuli even in principle, as the latter have no low-dimensional representation and, furthermore, the retinal responses are nonlinear. In the second approach, one first builds a probabilistic encoding model, followed subsequently by model-based inference of the most likely stimulus given the observed neural responses [15–21]. Theoretically, this procedure is possible for any stimulus, but in practice model inference is feasible only if it incorporates strong dimensionality reduction assumptions (e.g., that neurons respond to a linear projection of the stimulus). Here we demonstrate a third alternative, where a complex and dynamical stimulus is reconstructed from the output of the mammalian retina directly, by means of large-scale nonlinear regression. Retina is an ideal experimental system for such a study, because it permits stable recordings from large, diverse, local populations of neurons under controlled stimulation, where even simultaneous neural spiking events can be sorted reliably [22].

We start by performing linear decoding from the entire recorded retinal ganglion cell population, to separately reconstruct the temporal light intensity trace at each spatial location in the stimulus movie. When using sparse regularization, we extract and subsequently analyze “decoding fields,” the decoding counterpart of the cells’ receptive fields. We next examine nonlinear decoding using kernel ridge regression (KRR [23]) and deep learning [24], which provide a substantial increase in performance over linear decoding, and isolate spike train statistics that the nonlinear decoders are making use of. We conclude by examining how these statistics arise in generative models of spike trains and suggest that they might be essential for separating stimulus-driven from spontaneous activity.

## Materials and methods

### Data

Retinal tissue was obtained from adult (8 weeks old) male Long-Evans rat (Rattus norvegicus) and continuously perfused with Ames Solution (Sigma-Aldrich) and maintained at 32 °C. Ganglion cell spikes were recorded extracellularly from a multi-electrode array with 252 electrodes spaced 60 *μ*m apart (custom fabrication by Innovative Micro Technologies, Santa Barbara, CA). Experiments were performed in accordance with institutional animal care standards. The microelectrode covered a total retinal area of ∼ 1 mm^2^. For the rat this corresponds to 16-17 degrees of visual angle [25]. The spike sorting was performed with an in-house method based on [22].

### Visual stimulus

The stimulus movie consisted of randomly moving dark discs (*r* = 100 *μ*m) against a bright background (100% contrast, 2 ⋅ 10^12^ photons/cm^2^/s). The discs followed mutually avoiding trajectories generated through an Ornstein-Uhlenbeck process of the form:

$$\begin{array}{c} \frac{\Delta v_{i}}{\Delta t}=-\frac{1}{\tau }v_{i}+f_{i}+\sigma dW, \end{array}$$ (1)

where **v**_*i*_ is the velocity of the disc *i*, Δ*t* = 0.01 is the integration timestep, *τ* = 0.8 is the damping time constant, *σ* = 0.5 is the random force magnitude, *dW* is a zero-mean, unit-variance Gaussian random variable, and **f**_*i*_ is the hard-core central repulsive force between the discs and between each disc and the frame bounding box, with a decay of ∝ *r*^−6^, where *r* is the distance between the disc centers or between the disc and closest point of the bounding box. The resulting distribution of disc speeds peaked at *v* ≈ 0.6 *μ*m/ms and had a width of about *σ*_*v*_ ≈ 0.4 *μ*m/ms. The discs covered the recorded area uniformly to a very good approximation, with occupancy deviations at different encoding sites of ∼ 3%.

The movie was divided in segments of 1, 2, 4 and 10 discs, each 675 s long. Segments with increasing number of discs were presented sequentially and in total 3 segments of each type were shown, amounting to a total experiment time of 135 min. Each segment was regularly interspersed with 18 short (7.5 s) clips of repeated stimulus: in sum, 54 repeated clips were shown for each stimulus with different number of discs. The stimulus was convolved with a bank of 400 spatial symmetric gaussian filters (*σ* = 66.67 *μ*m) placed in a regular square 20x20 grid with *d* = 53*μm* spacing to produce local luminance traces. The filter normalization ensures the resulting traces are bounded in (0,1). The width of the filters was selected in preliminary tests to optimize decoding performance; specifically, in preliminary tests we found that filter widths in range *σ* ∼ 50 − 100*μm* minimized the mean-squared-error of L1-regularized linear decoders trained for a subset of decoding sites. The movie stimulus was shown at a refresh rate of 80 Hz. The response spike trains were binned accordingly in bins of 12.5 ms, and time aligned to the stimulus. The spatio-temporal receptive fields of the retinal ganglion cells were obtained through reverse correlation to a flickering checkerboard stimulus. The checkerboard was constructed from squares of 130 *μ*m that were randomly selected to be black or white at a rate of 40 Hz. Retinal spontaneous activity was recorded in full darkness (blackout condition) for 2.5 min.

### Linear decoder

Let $\vec{y}$ be a one-dimensional stimulus trace of length *N* time bins. In the linear decoding framework we assume that an estimate of the stimulus $\hat{\vec{y}}$ can be obtained from the neural response Σ as $\hat{\vec{y}}=\Sigma \cdot \vec{L}$, where $\vec{L}$ is a linear filter. In this formulation, the response of the retina is represented by the matrix $\Sigma \in R^{N\times (C\times \Delta T+1)}$, where *C* is the number of cells and Δ*T* the size in bins of the time window we associate with a single point in $\vec{y}$ (for all analyses Δ*T* = 61 corresponding to a window stretching from -375 ms to 375 ms around the time bin of interest, i.e., the decoding is performed using “acausal” filters). The extra dimension is a column of ones to account for the bias term in the decoding. Thus, the decoding filter $\vec{L}$ is structured as $\vec{L}=\left[L_{0}{\vec{L}}_{1}{\vec{L}}_{2}{\vec{L}}_{3}\ldots {\vec{L}}_{C}\right],$ where ${\vec{L}}_{i}$ is the filter corresponding to cell *i* and *L*_0_ is the bias term. We learned the filters $\vec{L}$ by minimizing the square error function with L1-regularization

$$\begin{array}{c} \chi^{2}=\frac{1}{N}{\left(\hat{\vec{y}}-\vec{y}\right)}^{⊤}\left(\hat{\vec{y}}-\vec{y}\right)+\lambda {\left\|\vec{L}\right\|}_{1}. \end{array}$$ (2)

To solve the minimization problem computationally we made use of the Lasso algorithm with the routines by Kim et al. [26]. Data was divided into training and testing sets (4.9⋅10^4^ training points, 2.3⋅10^4^ testing points). The filters were obtained from the training set and all measures of performance refer to the testing set. Regularization parameter λ was chosen through 2-fold cross-validation on the training set. The regularization term ensures the sparsity of the filters. Due to this sparsity some cells have negligible filter norms and therefore do not contribute to the decoding. This allows us to establish a hierarchy of cells by sorting them according to their filter norm $\|{\vec{L}}_{i}{\|}_{1}$. “Single-best cell” for every site refers to the cell with the largest norm. “Contributing cells” are the subset of cells with largest norm that jointly account for at least half of the total filter norm $\sum_{i}{\left\|{\vec{L}}_{i}\right\|}_{1}$.

All the results in the paper use acausal filters. If the decoding filters are restricted to be causal, the decoding performance can be significantly decreased; for test sites where we explored this effect, the cross correlation between true and linearly decoded luminance trace could decrease from ≈ 0.8 to ≈ 0.6. Given that our stimulus is a stochastic process and retinal processing necessarily entails some processing delay, this is not surprising.

### Kernelized nonlinear decoder

If instead of L1-regularization we enforce L2-regularization, the linear decoding filters can be obtained analytically through the normal equation

$$\begin{array}{c} \vec{L}=\Sigma^{⊤}{\left(\Sigma \Sigma^{⊤}+\lambda I\right)}^{-1}\vec{y}. \end{array}$$

Thus, an estimate of the stimulus for some new data $\hat{\Sigma }$ is given by

$$\begin{array}{c} \hat{\vec{y}}=\hat{\Sigma }\cdot \vec{L}=\hat{\Sigma }\Sigma^{⊤}{\left(\Sigma \Sigma^{⊤}+\lambda I\right)}^{-1}\vec{y}. \end{array}$$

Since this expression only depends on products of spike trains, we can make use of the kernel trick and substitute the usual scalar product by some appropriate nonlinear function *k* of the spike trains. In this way, we can express our nonlinear decoding problem as

$$\begin{array}{ccc} \hat{\vec{y}} & = & \kappa^{⊤}{\left(K+\lambda I\right)}^{-1}\vec{y}, \end{array}$$ (3)

$$\begin{array}{ccc} \kappa_{ij} & = & k({\hat{\vec{\sigma }}}_{i},{\vec{\sigma }}_{j}), \end{array}$$ (4)

$$\begin{array}{ccc} K_{ij} & = & k({\vec{\sigma }}_{i},{\vec{\sigma }}_{j}), \end{array}$$ (5)

where ${\vec{\sigma }}_{i}^{⊤}\in R^{1\times (C\times \Delta T+1)}$ is the *i*th row of matrix Σ. This is known as Kernel Ridge Regression [27, 28]. For our analyses we have used the Gaussian kernel

$$\begin{array}{c} k\left({\vec{\sigma }}_{i},{\vec{\sigma }}_{j}\right)=\text{exp}\;(-\frac{1}{2s^{2}}{\left\|{\vec{\sigma }}_{i}-{\vec{\sigma }}_{j}\right\|}_{2}^{2}). \end{array}$$ (6)

Before computing the kernel, it is customary to turn the spike trains into smooth traces for the sake of performance [29]. We convolved our spike trains with a Gaussian filter of 3 time bins width. The data was divided into training and testing sets (9.8⋅10^3^ training points, 2.3⋅10^4^ testing points). The parameters *s* and λ were obtained through joint 3-fold cross-validation on the training set. Since decoding at different sites is independent, *s* and λ were chosen separately at each site (likewise, L1 regularization strength for the linear decoder was also selected independently for each site). The performance of the nonlinear decoder depends on the set of cells considered. Contrary to the linear case where L1-regularization can effectively silence cells by setting their filters to zero, this nonlinear framework cannot ignore cells in a similar way. Therefore, including in the analysis non-informative cells can decrease the generalization performance of the decoder. To determine the best subset of cells for decoding we took advantage of the hierarchy of cells established by the linear L1-regularized decoding. We trained nonlinear decoders with progressively more cells (best cell, best two cells, etc.) and selected the subset of minimum decoding error on the training set (S7 Fig). Effectively, we jointly cross-validated the three parameters *s*, λ, and the subset size.

### Deep neural network

We trained a deep neural network on the decoding task. The architecture of the network is as follows: there are 5460 inputs (activity of 91 cells and 60 time bins) and two or three fully connected hidden layers followed by a fully connected linear output layer of 400 cells (corresponding to the 20x20 grid), see S20 Fig. The hidden layers have each 150 units with tanh activation function.

The loss is L2 loss on the regression error and L1 and L2 regularization on the weights (Elastic Net type), more specifically:

$$\begin{array}{c} \chi^{2}\left(\theta \right)=\frac{1}{N}\sum_{i=1}^{N}{\left({\hat{y}}_{\theta }\left(x_{i}\right)-y_{i}\right)}^{2}+\sum_{k=0}^{K}\lambda (|W^{\left(k\right)}{|}_{1}+|W^{\left(k\right)}{|}_{2}) \end{array}$$ (7)

where ${\hat{y}}_{\theta }\left(x_{i}\right)$ is the network output for input *x*_*i*_ and *W*^(*k*)^ is the weight matrix to layer *k* + 1, $\theta ={\left\{W^{\left(k\right)}\right\}}_{k=0}^{K}$. The regularization improves generalization by making the network weights smaller and creating a sparse connection graph (increases robustness to training set variations). The available data of 126360 input-output pairs was split into 79560, 23400, and 23400 points for training, validation and testing respectively (same test set as for other methods). We trained for 2500 epochs (each epoch trains on all training points once). To avoid that early during training many weights become zero because of the regularization we set λ = 0 for the first 100 epochs. We performed model selection by a grid search through the following hyperparameters: regularization constant: λ ∈ {5 ⋅ 10^−7^, 7.5 ⋅ 10^−7^, 1 ⋅ 10^−6^, 2.5 ⋅ 10^−6^, 5 ⋅ 10^−6^}, number of hidden layers *K* ∈ {2, 3}, and optimization method ∈ { stochastic gradient decent (sgd) with learning rate 0.01 and momentum of 0.9, Adam optimizer [30] with learning rate 0.005 and *ϵ* = 0.0001}. The hyperparameter setting with the smallest validation error where selected, resulting in: *K* = 3, λ = 2.5 ⋅ 10^−6^, and sgd.

Interestingly, only around 42 units per hidden layer have non-zero connections after training. Although, if started with only 50 units we observed worse performance. The mean of the square test error (over locations) is 0.01387 with standard deviation 0.00304.

### Classifiers

For classification purposes we assign each time bin to one of two classes: “fluctuating” or “constant”. “Fluctuating” corresponds to discs moving over the site of interest and decreasing the light intensity in that site, while “constant” refers to the constant illumination of the site when no discs are present. To label the time bins we use a simple cut-off criterion plus two further correcting steps to account for retinal adaptation effects. First we label as “fluctuating” every bin with stimulus intensity less than 0.99. Then we apply these corrections: *i)* Every identified “constant” segment shorter than 30 bins (375 ms) is relabelled as “fluctuating,” and *ii)* The first 30 bins following a “fluctuating” segment are also labelled “fluctuating.” In this way the stimulus at each site is divided in segments of fluctuating and constant intensity. We train both linear and nonlinear Support Vector Machine (SVM) classifiers to determine, from the spike train response, whether a given time bin is labelled as “constant” or “fluctuating”. Similarly to the decoding framework, to classify a given bin we consider a time window of Δ*T* = 61 bins around it in the response. For the nonlinear SVM we use the same gaussian kernel as in nonlinear decoding and the parameter values obtained when training the decoder. Note that this is not the optimal nonlinear classifier but allows us to evaluate the classifying power of the decoding kernel.

### Measures of performance

Given a stimulus intensity trace $\vec{y}$ and the corresponding decoding prediction $\hat{\vec{y}}$ we define the decoding error as the Mean Squared Error $\text{MSE}=N^{-1}{\left(\hat{\vec{y}}-\vec{y}\right)}^{⊤}\left(\hat{\vec{y}}-\vec{y}\right).$ We also make use of the related Fraction of Variance Explained defined as FVE = 1 − (MSE/Var(*y*)).

To measure decoding performance from the fully decoded movie we build Receiver Operating Curves (ROC). We threshold the decoded intensity trace at each site. If intensity is below threshold, the presence of a disc in the site is predicted. By comparing the prediction as a function of the threshold to the original stimulus frames (where the center of the site can only be white when no disc is present, or black when the disc is present), we can evaluate the performance of the decoder as a balance between the True Positive (TP) and False Positive (FP) rates

$$\begin{array}{c} \text{TPR}=\frac{\text{TP}}{\text{TP}+\text{FN}},\;\text{FPR}=\frac{\text{FP}}{\text{FP}+\text{TN}}. \end{array}$$

To assess the performance of the SVM classifiers we use the *F*_1_-score measure defined as

$$\begin{array}{c} F_{1}=2\frac{PR}{P+R}, \end{array}$$

where *P* is the Precision and *R* the Recall given by

$$\begin{array}{c} P=\frac{\text{TP}}{\text{TP}+\text{FP}},\;R=\frac{\text{TP}}{\text{TP}+\text{FN}}. \end{array}$$

For the binary classification task, “fluctuating” is defined as the positive class.

Unless otherwise stated, all of the statistical significance tests were performed with the Wilcoxon signed rank test.

### ON/OFF ratio bias estimation

For each site *s* we determine the set of available cells whose receptive field centers were less than 300 *μm* distant from the center of the site. We call *C*_*s*_ the total number of available cells at site *s*. In general, *C*_*s*_ is the sum of ON and OFF subtype cells, $C_{s}=C_{s}^{\text{on}}+C_{s}^{\text{off}}$. If, from the available cells at site *s*, we pick a random subset of size *N* = *N*^on^ + *N*^off^, the probability of choosing *N*^off^ cells is given by the hypergeometric distribution (random draw without replacement)

$$\begin{array}{c} p(N^{\text{off}}|s,N)=\frac{\left(\begin{array}{c} C_{s}^{\text{off}}\\ N^{\text{off}} \end{array}\right)\left(\begin{array}{c} C_{s}-C_{s}^{\text{off}}\\ N-N^{\text{off}} \end{array}\right)}{\left(\begin{array}{c} C_{s}\\ N \end{array}\right)}. \end{array}$$

The average probability over all sites considered is

$$\begin{array}{c} p\left(N^{\text{off}}|N\right)=\frac{1}{S}\sum_{s=1}^{S}p\left(N^{\text{off}}|s,N\right). \end{array}$$

Separately, for each site *s* we have established a hierarchy of cells from their decoding filter norms. Following the hierarchy we create decoding sets of different size *N* (the best cell, the best two cells, etc) and we count the number of OFF type cells *N*^off^ in them. We summarize this information in the histogram *M*(*N*^off^, *N*) that counts the number of sites where the decoding set of size *N* contains *N*^off^ OFF cells. With this histogram we obtain an empirical probability

$$\begin{array}{c} p_{\text{emp}}\left(N^{\text{off}}|N\right)=\frac{M(N^{\text{off}},N)}{S}, \end{array}$$

that we can compare with *p*(*N*^off^|*N*). In particular, the bias reported in S5 Fig is given by

$$\begin{array}{c} 100\cdot \frac{p_{\text{emp}}\left(N^{\text{off}}|N\right)-p\left(N^{\text{off}}|N\right)}{p(N^{\text{off}}|N)}. \end{array}$$

Only sites with *N*^off^, *N*^on^ ≥ 2 were considered for the comparison (n = 115).

### Encoding model

We build an encoding model for a single cell, based on the standard GLM type model proposed by Pillow et al [15]. The cell spikes stochastically through a Poisson process with a time-dependent firing rate λ(*t*) given by $\lambda \left(t\right)=f_{\alpha }\left((\vec{k}\vec{Y}\left(t\right)+\alpha \vec{h}\vec{\sigma }\left(t\right)\right)$ where $\vec{k}$ is a spatio temporal filter acting on stimulus $\vec{Y}$ and $\vec{h}$ is a temporal filter of the past spike history of the cell represented by $\vec{\sigma }$. The function *f*(*x*) is a rectifying nonlinearity of the log-exp form *f*(*x*) = *a* log(*b* exp(*x* + *c*)). The stimulus filter $\vec{k}$ factorizes into separate spatial and temporal filters. The spatial component is given by a balanced difference of gaussians, with widths *σ*_*c*_ = 35*μm* for the positive and *σ*_*s*_ = 100*μm* for the negative part, providing a symmetrical center-surround type filter. The temporal part of the filter is given by a single negative lobe of a sin-like function. The filter for the past spike history takes the form

$$\begin{array}{c} h\left(t\right)=A\;\text{sin}\left(t+\frac{\pi }{2}\right)\;\text{exp}\left(B\left(-t+\frac{\pi }{2}\right)\right). \end{array}$$

This filter inhibits firing after a spike but, depending on the values of the parameters, it can have a positive lobe after the inhibitory part that tends to increase the firing rate. We consider a span of 250 ms (20 bins) for both the past history filter and the temporal part of the stimulus filter. All elements of the filter are fixed except for the rectifying nonlinearity that is changed according to the value of *α*. Initially, the parameters of the nonlinearity *f*_*α* = 1_(*x*) are adjusted to provide an average firing rate similar to that observed in real data. The *α* = 1 model is taken as the ground-truth and every time *α* changes, the nonlinearity *f*_*α*_(*x*) is fitted anew by maximizing the likelihood on *α* = 1 rasters, in order to reproduce the firing rate trace (PSTH) as closely as possible to the PSTH generated by *α* = 1. The model neuron is stimulated with real data and the intensity trace at the central site of its receptive field is the stimulus considered for decoding. The model has been implemented using the Nonlinear Input Model toolbox [31].

## Results

### Decoding setup

**Fig 1:**
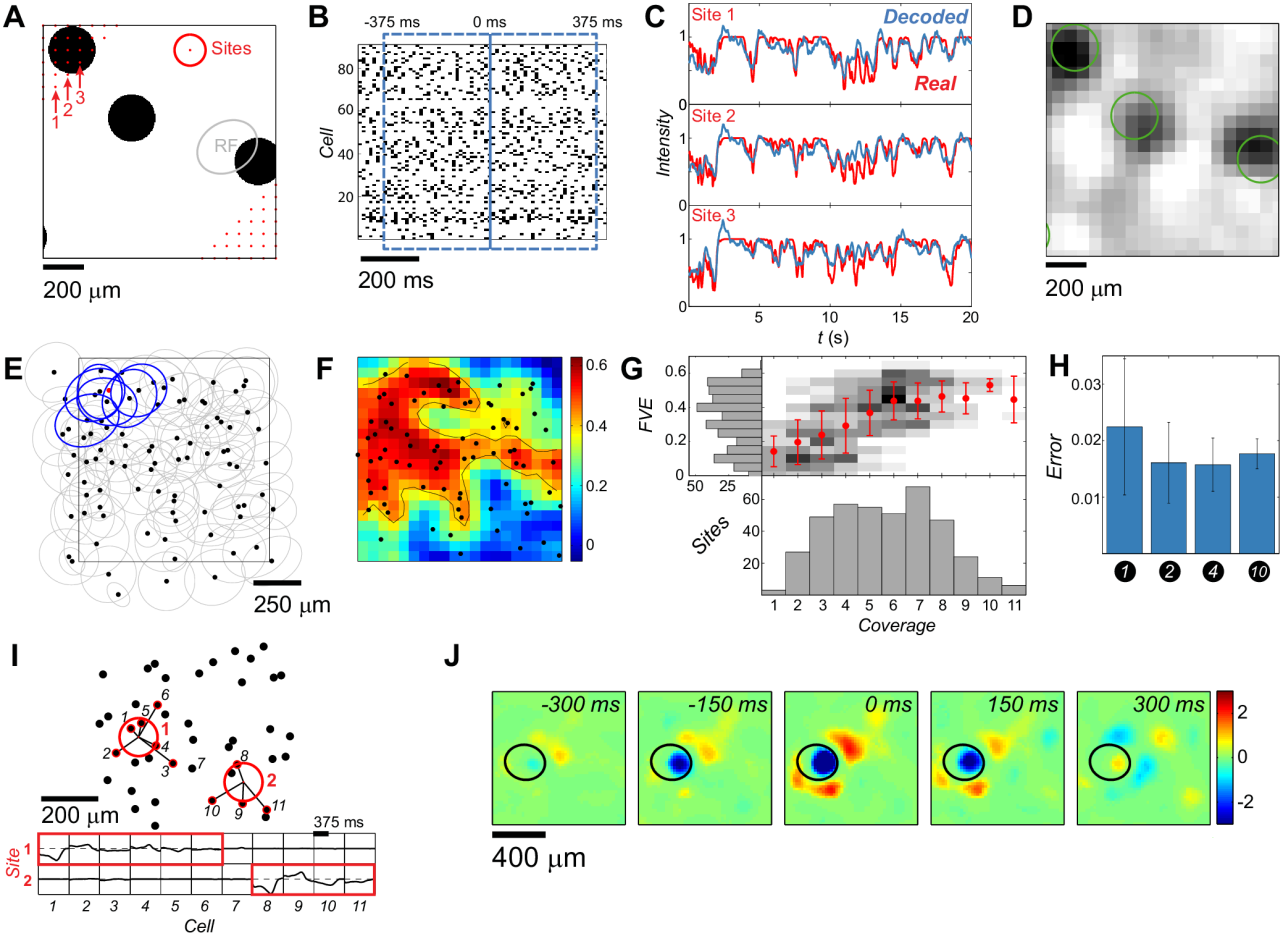
Linear decoding of a complex movie. **A**: An example stimulus frame. At each site (red dots = partially shown 20×20 grid) the stimulus was convolved with a spatial gaussian filter (red circle = 1*σ*). Typical RGC receptive field center size shown in gray. **B**: Responses of 91 RGCs with 750 *ms* decoding window overlaid in blue. **C**: Three example luminance traces (red) and the linear decoders’ predictions (blue). **D**: Decoded frame (same as in **A**) reconstructed from 20×20 separately decoded traces. Disc contours of the original frame shown for reference in green. **E**: RF centers of the 91 cells (black dots = centers of fitted ellipses). RF centers overlapping a chosen site (red dot) are highlighted in blue. **F**: Performance of the linear decoders across space, as Fraction of Variance Explained (FVE). Black dots as in **E**; black contour is the boundary *FVE* = 0.4. **G**: Performance of the linear decoders (FVE) across sites as a function of cell coverage (grayscale = conditional histograms, red dots = means, error bars = ± SD). **H**: Average decoding error across sites (MSE ± SD) of 10-disc-trained decoders, tested on withheld stimuli with different numbers of discs. **I**: Cells (black dots = RF center positions) contributing to the decoding at two example sites (red circles); decoding filters shown below. For each site, contributing cells (highlighted in red and joined to the site) account for at least half of the total L1 norm. **J**: Decoding field of a single cell (here, evaluated over a denser 50×50 grid and normalized to unit maximal variance); the cell’s RF center shown in black.

We recorded the spiking activity of *C* = 91 ganglion cells from a 1 mm^2^ patch of the rat retina, while presenting a complex and dynamical stimulus that consisted of 1, 2, 4 or 10 black discs on a bright background (Fig 1A and Methods). The discs followed self-avoiding random motion, generated as described in the Methods section, which (for decoder training) was non-repeated; all decoding results are reported on withheld (test) segments of the stimulus that were not used during training. The stimulus also contained a segment of repeated trajectories that was randomly interspersed into the non-repeated part and used only to assess the role of noise correlations. Our goal was to reconstruct the light intensity as a function of time at a grid of 20 × 20 spatial positions (“sites”) uniformly tiling the stimulus frame. Specifically, at each site, we convolved the original movie with a small Gaussian filter (see Methods), which defined the “luminance trace” at that site, to be decoded. Stimulus features (here, disc size) were smaller than the receptive field center of a typical recorded RGC, making the decoding task non-trivial.

### Sparse linear decoding of a complex movie

To estimate the luminance trace at any given time, we trained a separate sparse linear decoder for each site on a 750 ms sliding window of the complete spiking raster, shown in Fig 1B, and represented as spike counts in Δ*t* = 12.5 ms time bins (see Methods). The decoder minimized the square error between the true and estimated luminance trace at each site, using sparse (L1) penalty on decoding weights, as specified by Eq (2). While each decoder in principle had access to all neural responses, the sparse penalty ensured that the majority of the weights corresponding to redundant or non-informative neural responses for each site were zeroed out, yielding interpretable results which we describe in detail below. When trained on the 10-disc stimulus, this procedure predicted well the luminance traces across individual sites on withheld sections of the stimulus (Fig 1C), allowing us to reconstruct the complete movie (Fig 1D).

We expected the performance of our decoder to depend strongly on local coverage, i.e., on the number of recorded cells whose receptive field centers overlap a given site. Coverage amounted to about six cells on average and exhibited substantial spatial heterogeneity, as shown in Fig 1E. The quality of our movie reconstruction, measured locally by “fraction of variance explained” (FVE, see Methods), showed similar spatial variation (Fig 1F) which correlated strongly with coverage (Fig 1G), and saturated at ≥ 6 cells. In what follows, we restrict our analyses to sites with good coverage that pass a threshold of FVE ≥ 0.4. Despite the high dimensionality of this regression problem (decoders have ∼5 ⋅ 10^3^ parameters per site), sparse regularization ensured uniformly good performance even when tested on out-of-sample stimuli with varying number of discs (Fig 1H).

To analyze how rich stimuli are represented by a population of ganglion cells with densely overlapping receptive fields, we examined the resulting decoding weights in detail. We found that stimulus readout was surprisingly local. As illustrated for two example sites in Fig 1I, only a few cells whose receptive field centers were in close proximity to the respective sites were assigned non-negligible decoding weights. This was true in general: on average 5.4 ± 2.8 cells, whose RF centers were all located within 200 *μm* of the decoded site, contributed to the luminance trace reconstruction; cells beyond this spatial scale contained no decodable information (S1 and S2 Figs).

Our framework also allowed us to construct a “decoding field” for every cell (Fig 1J). A decoding field represents an impulse response of the decoder, i.e., an additive contribution to the stimulus reconstruction for every spike emitted by a particular cell. While one can reasonably expect that the receptive and decoding fields overlap in location and spatial extent, there is no theoretical guarantee that this must happen, given that neural encoding is strongly nonlinear. We nevertheless confirm this expectation and observe a very good correspondence between the spatial locations and sizes of the decoding and receptive fields for all cells (S3 Fig), with decoding fields also exhibiting a clear center-surround-like structure. We find that decoding filters shapes for all cells are highly stereotyped (S4 Fig). We further find that the readout of retinal responses is local, in the sense that only cells with receptive field centers close to the decoding site contribute to the decoding (Fig 1I, S6 Fig). Lastly, the readout is structured, in the sense that the cells that contribute to decoding at each site have a preferred ON vs OFF composition that favors recruiting OFF cells (S5 Fig), most likely because the visual feature that moves in our stimulus is a dark disc on a bright background.

Taken together, our results suggest that retinal responses to complex stimuli can be read out in a highly stereotyped, structured, and local manner.

### Nonlinear decoding outperforms linear decoding

Could nonlinear decoding improve on these results? We considered two nonlinear regression methods that can tractably be applied to our data: kernel ridge regression (KRR) and regression using deep neural networks.

Kernel ridge regression is a well-known extension of linear regression into the nonlinear domain by means of the kernel trick. We used Gaussian kernels whose width was determined using cross-validatation (see Methods) [29], as specified in Eqs (3)–(6). Importantly, the success of this nonlinear decoder crucially depended on the proper selection of local groups of cells relevant for each site, as identified by linear decoding: its sparse (L1) regularization acted as “feature selection” for the nonlinear problem (Methods, S7 Fig). Nonlinear decoder could then make use of higher-order statistical dependencies within and between the selected spike trains to achieve high performance.

We compared these results to regression using neural networks. An architecture that achieved good performance consisted of an input layer (that received spiking rasters of the same dimension as the linear regression problem), followed by three fully-connected hidden layers with 150 sigmoidal neurons each, followed by a 20 × 20 output layer whose units correspond to the decoding sites of our movie; this architecture is schematized in S20 Fig. The network was trained by minimizing the squared reconstruction error, Eq (7), using standard deep learning tools (see Methods).

**Fig 2:**
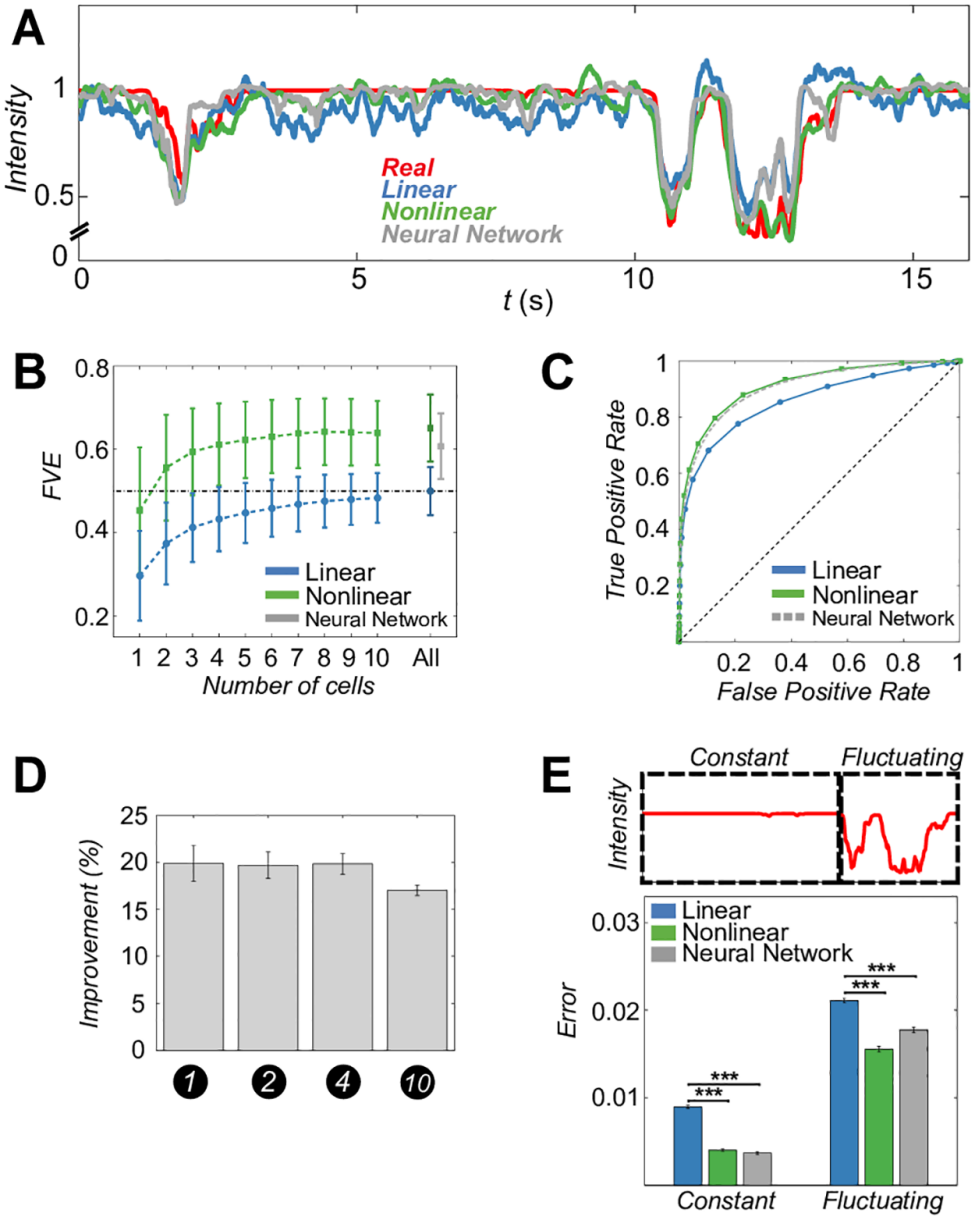
Nonlinear decoding outperforms linear decoding. **A**: Luminance trace (red) with linear (blue) and nonlinear KRR (green) and neural network (grey) predictions. **B**: Average decoder performance (± SD across sites), achievable using increasing numbers of cells with highest L1 filter norm. For nonlinear decoding, “All” is the optimal subset that maximizes performance (S7 Fig). Since the neural network (grey point with an error bar) simultaneously decodes the movie at all sites, it only makes sense to train it using “All” cells. **C**: Average ROC across all testing movie frames. **D**: Fractional improvement (average ± SEM across sites) of nonlinear KRR versus linear decoders for test stimuli with different numbers of discs. All decoders were trained only on the 10-disc stimulus. **E**: Decoding error (MSE; average ± SEM across sites) in fluctuating and constant epochs is significantly larger for linear decoders (p<0.001) relative to nonlinear KRR and the neural network.

Fig 2A shows a luminance trace at one of the example sites, together with its linear and nonlinear reconstruction. Nonlinear decoders track better the detailed structure of luminance troughs, which occur when discs cross the site, as well as exhibiting smaller fluctuations when no discs are crossing the site and the true luminance trace is therefore constant. This is reflected in a substantial overall increase in fraction of variance explained (FVE) across different sites, shown in Fig 2B. A kernelized nonlinear decoder using only two best cells per site outperforms, on average, the best sparse linear decoder constructed from the entire population; nonlinear performance saturates quickly with the number of cells and peaks when decoding from local ∼8-cell groups. The neural network decoder, which we train on the complete neural population, reaches a comparable performance to the best kernelized decoder.

An alternative way to compare decoding performance is to threshold the sequence of decoded movie frames (see S8 Fig and S1 Video), thereby assigning each site to a decoded dark disc (“below threshold”) or to the bright background (“above threshold”). Decoded movie frames can then be compared to ground truth (i.e., the original movie frames which can only be either black or white at every location) at each threshold using the receiver operator characteristic (ROC curve), shown for all decoders in Fig 2C. In this metric, the performance of the kernelized and neural network decoders are nearly indistinguishable, and consistently outperform linear decoders. Excess nonlinear performance of between 15 and 20% of FVE was maintained even when decoders were trained on 10-disc stimulus and tested on stimuli with smaller number of discs (Fig 2D). Excess nonlinear performance was also observed when decoding from a cell mosaic of a single functional type (S9 Fig) and on a repeat experiment (S10 Fig).

We note the surprising consistency between the kernelized decoding and neural network results. Despite the fundamental differences in the nature and application of these two regression methods—neural networks are universal approximators, use different regularization from the kernelized decoders, and have been trained on all cells simultaneously to decode at all sites simultaneously, in contrast to the kernelized decoders—their numerical measures of performance appear quantitatively consistent. While it is impossible to exclude the possibility that yet another type of decoder could yield much higher performance, it is also possible that both nonlinear decoders we tried managed to extract all available information about local luminance traces from the recorded spike trains.

Another particularly striking feature of our results was the difficulty of the linear decoder to match the true (constant) luminance trace when no disc was crossing the corresponding site. Rat retinal ganglion cells are continuously active even when there are no coincident on-center luminance changes, with the activity likely resulting from stimulus changes in the surround, from long-lasting sustained responses to previous stimuli, from effective network coupling to cells that do experience varying input, or from true spontaneous excitation that would take place even in complete absence of stimuli [32–35]. Either way, activity of cells at constant local luminance presents a confound that is difficult for a generic linear mechanism to eliminate, which results in decoder fluctuations, or “hallucinations,” of sizeable variance. To quantify this effect, we partitioned the luminance traces at every site into constant and fluctuating epochs by means of a simple threshold (see Methods), and examined decoding errors separately during both epochs. The decrease in decoding error by using nonlinear decoders was similar in absolute terms in both epochs, but represented a much larger fractional decrease during constant epochs, suggesting that nonlinear decoders might specifically be better at suppressing their responses to spontaneous-like neural activity (Fig 2E).

We reasoned that this improvement comes, in part, from the ability of both nonlinear methods to recognize whether there are any on-site luminance fluctuations or not, from the spike trains alone. To test this idea, we trained linear and nonlinear kernelized classifiers, operating on identical inputs and with the same kernel parameters as the decoders, to best separate constant from fluctuating activity. Consistent with our expectations, nonlinear classifiers outperformed linear at every site, irrespectively of whether their input were the rasters of all local cells that contribute to the decoding, as shown in S11A Fig, or the raster of a single best cell at every site, as shown in S11B Fig.

### Nonlinear decoders make use of spike-history dependencies in individual spike trains

Next, we attempted to identify the statistics of spike trains that are necessary to explain the excess performance of nonlinear decoders. Our starting point was the following observation: the simplest nonlinear decoders that used a single best cell for each site, when interrogated with a test-set epoch of pure spontaneous activity (i.e., neural responses to a completely blank screen), yielded luminance traces with significantly smaller variance than their linear counterparts (S12 Fig). Since the only structure in spike trains during spontaneous activity is, by definition, due to “noise correlations”—pairwise or higher-order dependencies between spikes within an individual spike train or across different spike trains—we hypothesized that certain noise correlations could be used by nonlinear decoders also during stimulus presentation to boost their decoding performance.

**Fig 3:**
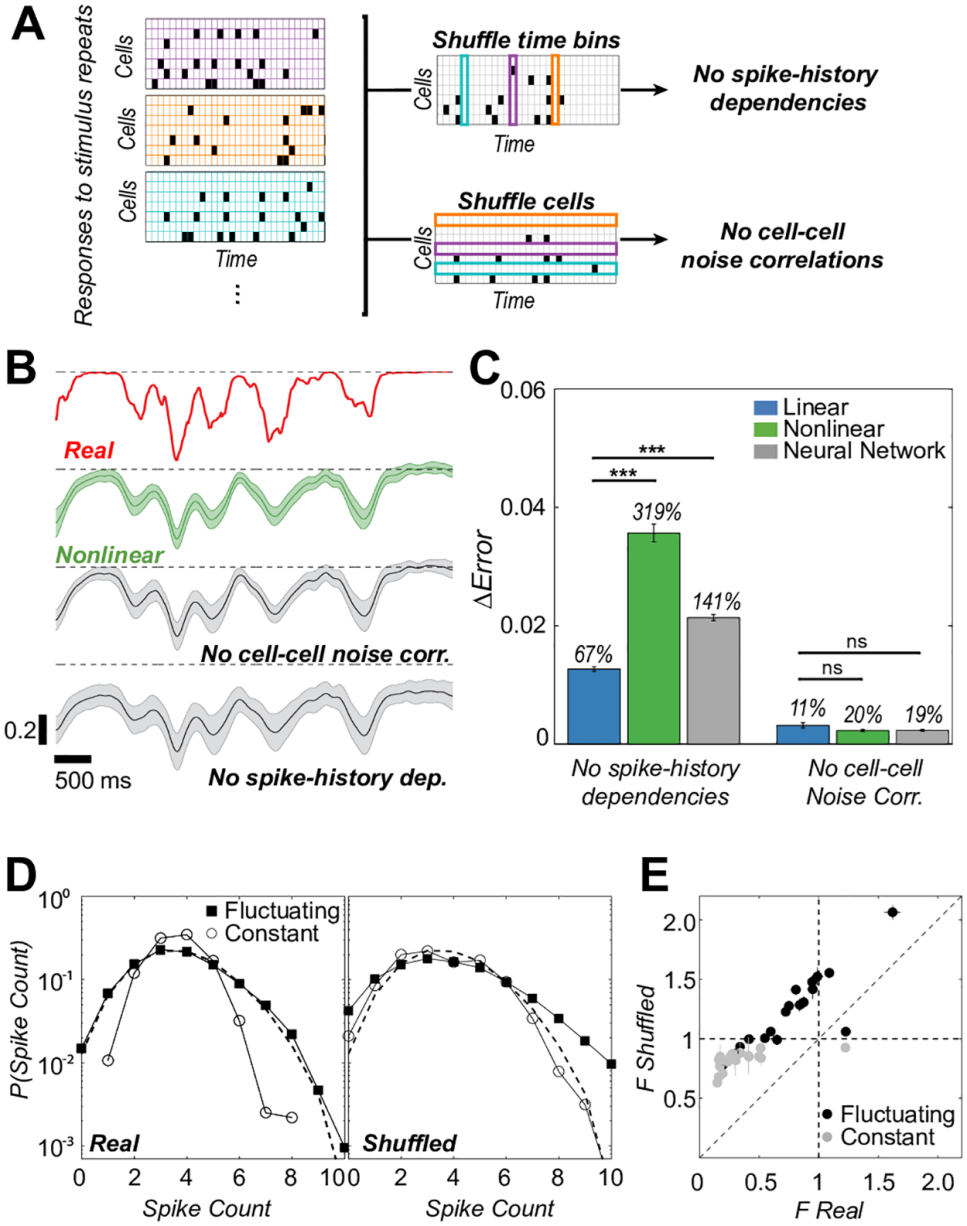
Spike-history dependencies affect decoding performance. **A**: Shuffles of responses to repeated stimulus presentations remove different types of correlations, but preserve average locking to the stimulus (PSTH), and thus stimulus-induced correlations. **B**: A repeated stimulus fragment (red trace), nonlinear kernelized decoder predictions using real responses (green), and using responses without different types of correlations (gray); shown is the prediction mean ± SD over repeats. **C**: Increase in decoding error (MSE) when spike-history dependencies or noise correlations are removed (average ± SEM across sites); percentages report fractional differences relative to the original performance. **D**: Spike count distributions for a single example cell. Removing spike-history dependencies broadens the distributions, in particular in constant epochs. Dashed line = expectation for a fully randomized spike train with a matched firing rate. **E**: Variance-to-mean ratio *F* of spike count distributions for spike trains with and without spike-history dependencies. Each point is a cell that contributes most to decoding at a particular site (when the same cell contributes to multiple sites, average ± SD across sites is shown).

To test this hypothesis, we made use of many identical repeats of a particular stimulus fragment embedded in our disc movie (these repeats were used neither for training nor testing). Using the same decoders as above, we decoded the original response rasters corresponding to the repeated fragment, as well as rasters in which we shuffled the spikes to remove spike-history dependencies, or to remove cell-cell noise correlations, as shown in Fig 3A, to assess how decoding is impacted by the removal of certain types of correlation in the spike trains. Note that these manipulations left the firing rates of all cells intact, and thus preserved all correlations in the spike trains that are due to the neurons responding to a spatio-temporally correlated stimulus. Crucially, for our analysis we did not retrain our decoders on the shuffled spike trains, because we wanted to ask whether the *same* decoders that we trained to read the real (unshuffled) neural code can also read the modified neural code lacking various components of the noise correlations. If the decoder performance were unaffected by such removal, then noise correlations are not crucial for our decoder; in contrast, a drop in decoder performance would suggest that noise correlations may be necessary. Alternatively, if we were to retrain our decoders on the shuffled spike trains, we would be answering a different question: Is there *any* decoder that can read the shuffled neural code? While interesting, (i) it is unclear what statements about the actual neural code such an analysis would provide (since these decoders would be trained on synthetic, shuffled codes that only exist in our computer); (ii) technically, the number of distinct training samples would be drastically too small to train such decoders, since the experiments impose a hard trade-off between the number of repeats and the duration of the repeated fragment, on which the decoder would have to be trained. For these scientific and methodological reasons, we performed the following analysis using decoders trained on actual (unshuffled) responses to unrepeated stimuli.

Fig 3B shows a stimulus reconstruction at an example site by the nonlinear kernelized decoder, for original rasters as well as rasters with removed spike-history dependencies or cell-cell noise correlations. Removing cell-cell noise correlations leads to a small increase in the variance of the reconstructions across stimulus repeats, with only marginal differences in the mean reconstructed trace, compared to decoding from intact rasters. Surprisingly, removing spike-history dependencies leads to much worse reconstructions, whose mean is strongly biased and variance increased; as a result, the dynamic range of the decoded trace is substantially lower compared to decoding from intact rasters. These observations are summarized across sites in Fig 3C, which shows the increase in decoding error when spike-history dependencies or cell-cell noise correlations are removed. Removal of cell-cell noise correlations leads to small increases in error, roughly of the same magnitude for both linear and nonlinear decoders; in contrast, while removal of spike-history dependencies leads to increases in error for both decoders, the effect is two-to-three-fold larger for the two nonlinear methods. We emphasize that kernelized decoders and neural network are two fundamentally different regression methods, yet the removal of spike-history dependences strongly decreases the performance of both, suggesting that our observations are likely not a consequence of choosing a particular decoder type. Qualitatively similar conclusions hold for the classifiers trained to separate constant from fluctuating input epochs (S13 Fig), as well as for decoders and classifiers trained on the single best cell per site (S14 Fig).

Having established that spike-history dependencies are crucial to the performance of the nonlinear decoders, we looked at the detailed statistical structure of individual spike trains. For each neuron that best decoded the luminance trace at a specified site, we focused on 250 ms (20 time bins) response sequences and constructed a distribution over the number of occupied time bins (“spike counts”), separately for epochs where the luminance trace was fluctuating or where it was constant. As shown in Fig 3D, these distributions differed significantly: the count distribution was much tighter in constant epochs, while the mean firing rate between the epochs did not change much. During fluctuating-input epochs, observing more spikes in a 250 ms window was more likely than at constant input, but—perhaps surprisingly—patterns with very low numbers of spikes (e.g., zero or one) were also more likely during fluctuating-input epochs. The count distribution at fluctuating light was very similar to binomial (and, at this temporal resolution, Poisson), while it was tighter at constant light. These changes could be summarized by a simple statistic, the variance-to-mean ratio *F* = (variance in spike count)/(mean spike count). Note that unlike the standard Fano factor, our *F* is not computed across the repetitions of the same stimulus and thus measures the total variability in the response, which includes variance due to the changing stimulus. When we removed spike-history dependencies, the variance-to-mean ratio *F* increased for both distributions and they became harder to distinguish from each other. Fig 3E shows that this behavior was consistent across all sites, highlighting the very high regularity of neural spiking that resulted in sub-Poisson variance (*F* substantially below 1) during epochs of constant luminance.

How could spike-history dependencies help in stimulus decoding? A possible scenario would involve the situation where decoders should “sum” multiple spikes from the same neuron in the recent past super-linearly, to optimally reconstruct the stimulus. In this case, without spike-history dependencies that are responsible for precise firing with sub-Poisson variance, the Poisson spiking in the absence of spike-history effects would cause large (compared to linear decoder) spurious variance in the decoder output. Adding spike-history dependencies would, in contrast, tighten the number of emitted spikes, giving the nonlinear decoder a reliable option to sum spike effects super-linearly without being swamped by spiking noise. We emphasize that this is only the simplest scenario we could think of as an example where spike-history dependencies could be beneficial; there are likely many others.

Taken together, our results show that: *(i),* spike-history dependencies within individual spike trains are crucial for nonlinear decoder performance; *(ii),* these dependencies shape the distribution of spike counts on timescales relevant for decoding; *(iii),* during constant local luminance, spiking activity is very regular (and statistically similar to true spontaneous activity, see S15 Fig); *(iv),* a simple statistic, which summarizes the effects of spike-history dependencies in different epochs and their changes when the spike trains are shuffled, is the spike variance-to-mean ratio *F*. This does not imply that nonlinear decoders actually compute some version of a local estimate for *F*: they could be sensitive to other statistics, e.g., the interspike interval distribution, which also differs substantially between the epochs, see S16 Fig. Because nonlinear decoders we use have no well-defined set of sufficient statistics, it is impossible to claim which precise statistic of the spike train they are sensitive to, beyond stating that they clearly are sensitive to the removal of spike-history dependencies. Note further that we can only establish clearly that nonlinear decoders that we trained are sensitive to the removal of spike-history dependencies; we, however, cannot exclude the option that there exist nonlinear decoders of the same class that reach similar performance as ours but are at the same time robust to the removal of spike-history dependencies. Nevertheless, subsequent analyses on synthetic data that we provide below, as well as the robustness of our observations with respect to the nonlinear method (kernelized decoder and the neural network) suggest that crucial decoding information really is present in the spike-history dependencies, and that the underlying reason for nonlinear decoder performance is its ability to recognize high regularity of spiking during epochs of constant local luminance.

### A simple neural encoding model can recapitulate spike train statistics crucial for nonlinear decoding

**Fig 4:**
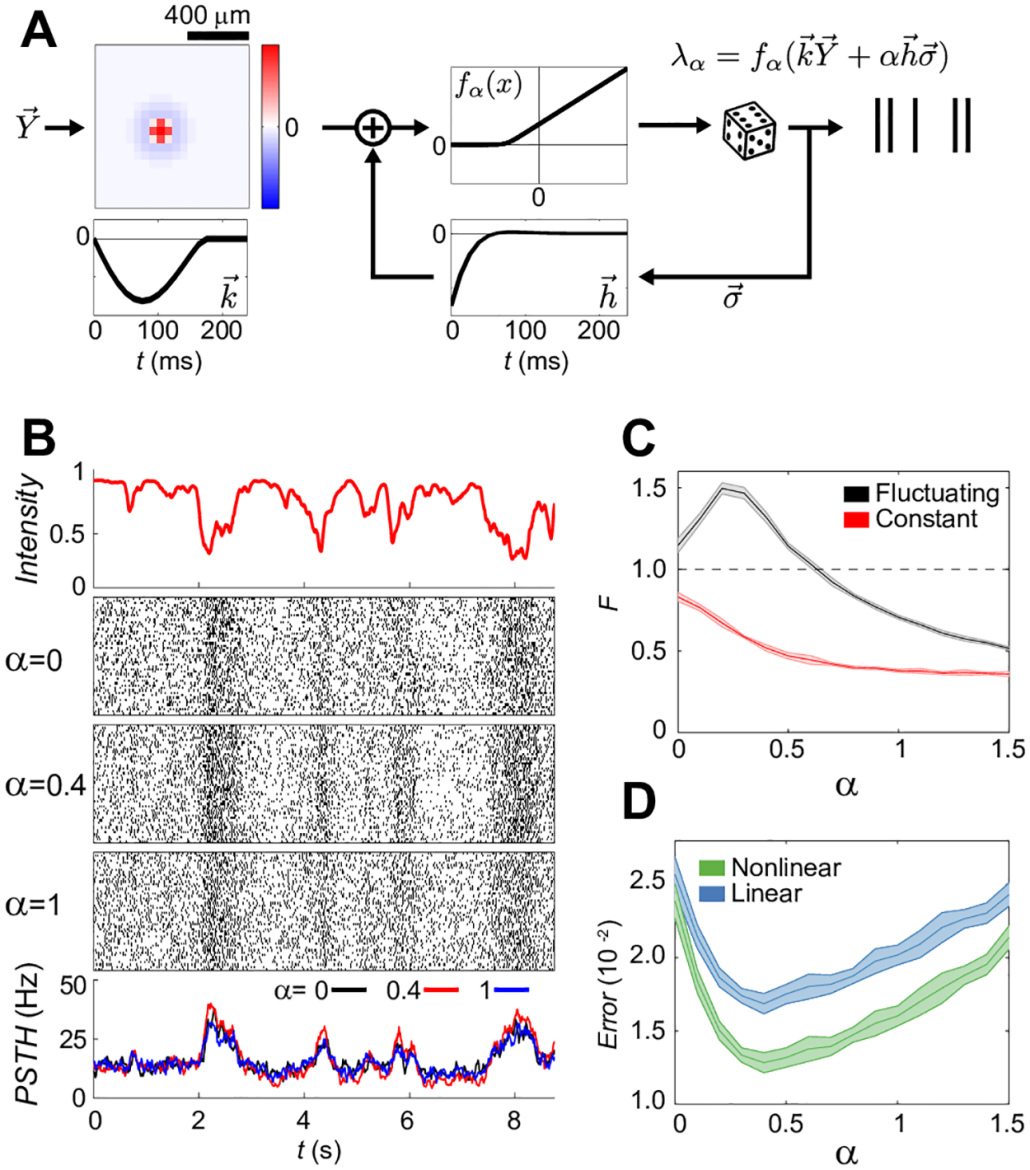
Spike-history dependencies of intermediate strength facilitate nonlinear decoding in simple models of neural processing. **A**: Schematic of a single-cell Generalized Linear Model (see Methods). The neuron’s sensitivity to the stimulus is determined by a radially symmetric difference-of-Gaussians spatial filter that has a monophasic timecourse ($\vec{k}$), and combines additively with the neuron’s sensitivity to its own past spiking, given by filter $\vec{h}$ (with strong refractoriness followed by weak facilitation). Importantly, $\vec{h}$ shapes spike-history dependencies in the resulting spike trains. A nonlinear function *f*(⋅) (here, threshold-linear) of the combined sensitivities gives the neuron’s instantaneous firing rate that can be used to generate individual spike train instances. Shapes, as well as the temporal and spatial scales of the filters, were realistic for our data. **B**: Example rasters (50 repeats) generated with the encoding model for a given intensity trace and different magnitudes (*α*) of spiking history filter $\vec{h}$. The rasters are matched in PSTH (bottom) but differ in temporal noise correlations. **C**: Average spike count variance-to-mean ratio, *F*, (± SD) of the model as a function of *α* in fluctuating and constant epochs. **D**: Decoding error as a function of *α*. Decoders are trained for each separate *α* and tested on withheld stimuli; shade = SD over 10 spike train realizations.

Can the observed spike-history dependencies, which enable successful nonlinear decoding, be generated by simple and generic neural encoding models? To address this question, we made use of generalized linear models (GLMs) [36, 37], probabilistic functional models of spiking neurons that extend the paradigmatic linear-nonlinear (LN) framework by incorporating the recurrent feedback from neuron’s past spiking, as schematized in Fig 4A. Previously, GLMs have been successfully applied to responses of the mammalian retina [15, 20] and in the cortex [38, 39], and also reproduced well the firing rates of cells recorded in our experiment on the repeated stimulus fragment (S17 Fig).

To link encoding models and decodability in a way that would generalize beyond the specifics of our dataset, we created the simplest stereotyped model cell, shown in Fig 4A. Crucially, we parametrized the magnitude of the self-coupling filter with *α*: *α* = 0 thus corresponded to a pure LN model, while increasing values of *α* made neural spike trains non-Poisson, progressively enforcing dependence on past spiking and consequently increasing the magnitude of the resulting temporal correlations.

With this model in hand, we generated a “baseline” raster of repeated responses to a randomly moving disc stimulus at an initial value of *α* = 1, which corresponds to the strength of spike-history dependence inferred from our data, as shown in Fig 4B. The average firing rate was chosen to be the typical rate of our recorded ganglion cells. We then systematically changed the value of *α* and, for each value, refitted the nonlinearity by maximizing the likelihood to the baseline raster at *α* = 1 (see Methods). This procedure generated synthetic rasters that, to an excellent approximation, were matched in their peri-stimulus time histograms (PSTH) and stimulus preference, yet differed in the strength of spike-history dependencies.

Following our previous analyses, we partitioned the luminance trace into constant and fluctuating epochs, and looked at the spiking statistics in 250 ms (20 time bin) windows. Spike count variance-to-mean ratio *F* in constant epochs decreased as a function of *α* and dropped substantially below 1; in contrast, when on-center luminance was fluctuating, *F* behaved non-monotonically (Fig 4C). In line with expectations and behavior observed in our data, *F* at constant luminance was always below *F* at fluctuating luminance. Having ensured that the statistics of synthetic rasters qualitatively agreed with the data for the range of *α* we examined, we asked about the performance of linear and nonlinear decoders, trained and tested at different values of *α*. Fig 4D plots the decoding error as a function of *α*; see S19 Fig for the separation into error in fluctuating vs constant epochs as a function of *α*. Overall, the error levels are in range of those observed for real data (cf. Fig 1H), with nonlinear decoders outperforming linear by ∼10 − 30%. Interestingly, the minimal error for both decoders is achieved at an intermediate value of α* ≈ 0.4, which also corresponds to the point where nonlinear decoders maximally outperform their linear counterparts. At *α* = 0, where the encoding models are effectively LN neurons, the decoders differ only marginally in performance (analogous results hold for the classifiers separating fluctuating from constant epochs, see S18 Fig).

How close are real retinal ganglion cells to the value of *α* that permits best nonlinear reconstruction? This question cannot be answered precisely with the toy models we use. Within the class of generalized linear models (GLMs) considered here, we can reliably show that nonlinear decoding performance significantly outperforms linear decoding performance for a broad range of *α* values that includes both α* ≈ 0.4 as well as *α* = 1 (which, by our definition, corresponds to the best fit of GLM model to our data), and this effect is robustly true for all the cells that we examined. It is, however, likely that GLM models are too simplistic for the cells we are considering (realistic models for rat ganglion cells may require two nonlinear stages of stimulus processing, i.e., LNLN models [40]), if we wanted to make a quantitative statement about how close real cells are to the value of *α* that permits optimal nonlinear reconstruction. In these simulations we also haven’t modeled cell-to-cell noise correlations; further, it is likely that GLM does not capture all spike-history dependencies; and we decoded only the central pixel of the model’s receptive field. These differences between the simulations and the real experiment are likely responsible for the fact that the difference between nonlinear decoding performance from GLM-generated spike trains and same spike trains with shuffle-removed spike-history dependencies are much smaller than what we see in real data. Nevertheless, while the quantitative match between real neural data and GLM simulations is beyond the scope of this paper, we have shown qualitatively that in a generic class of encoding models that have been widely applied to both peripheral as well as central neural processing, there exists a non-trivial strength of spike-history dependence that facilitates nonlinear stimulus reconstruction. Intuitively, the existence of optimal α* > 0 can be explained as a trade-off between ensuring regularity of spiking during constant epochs, which the nonlinear decoder can make use of, while not impeding stimulus encoding during fluctuating epochs; during these epochs, stimulus-driven term should dominate over sensitivity to past spiking, otherwise excessive dependence on spiking history (e.g., *α* ≥ 1 in Fig 4B) could perturb reliable locking to the stimulus.

## Discussion

Insights from decoding provide crucial constraints for theoretical models of neural codes. A large body of work dissects nonlinearities in stimulus processing, from nonlinear summation in the receptive field or during adaptation, to essential spike generation nonlinearities. Consequently, one would expect nonlinear decoding to outperform linear, but reports to that effect are scarce [11, 41]. In theory the results of a nonlinear encoding process can be linearly decodable [42, 43], yet whether this is true of real neurons under rich stimulation is still unclear. What has been demonstrated to date is that certain low-level representations of simple stimuli—but not the full frame-by-frame movie—can be linearly decoded [12, 44]. Another fundamental question concerns the stability of decoding transformations, which has recently received renewed attention in the context of efficient coding [45–47]. Finally, a number of studies, both theoretical [48] and data-driven [7, 15, 20, 49–51], focused on correlations in neural activity, especially those due to spike-history dependence and network circuitry (“noise correlations”); here, decoding provides a way to quantitatively ask about the functional contribution of such correlations to stimulus reconstruction. Approaching these issues empirically requires us to first construct high-quality decoders for complete stimulus movies—conceptually, doing the inverse of the state-of-the-art encoding models [15]—which remains an open challenge.

Some of the above questions have been approached before using frame-by-frame decoding, with stimuli of varying complexity. Theoretical methods for such decoding—as well as several approximations to render these methods tractable—have been presented, mainly in the context of probabilistic-model-based decoding [16–19], although they have generally not been applied to real recordings with rich stimuli. Linear decoding of natural scenes has been undertaken in the cat LGN with linear decoders (but without sparse regularization) [52], and using Bayesian methods with strong natural movie priors from fMRI recordings of the visual cortex [53]. Generalized linear models (GLMs) have been used to model the neural responses (e.g., [15, 20, 21]), although full stimulus reconstruction was undertaken only in [15] for a binary checkerboard stimulus, whereas other works used the inferred probabilistic models to perform the easier tasks of stimulus classification or decoding from synthetically generated spike trains. This is, in part, because optimal (Bayesian) decoding of stimuli with complex prior statistical structure (such as ours or natural movies) is technically challenging. Furthermore, for many neural systems, including but not limited to the retina under natural or complex dynamical stimulation, we do not have adequate encoding models; consequently, optimal Bayesian inversion of poor encoding models does not represent a clearly interpretable benchmark for other decoding methods. We thus decided for an alternative, statistical approach of constructing nonlinear decoders directly and benchmarking them against an accepted common standard, the linear decoder.

To this end, we used large-scale linear and nonlinear (kernelized, neural network) regressions to directly decode a complex stimulus movie from the output of many simultaneously recorded retinal ganglion cells. Importantly, we did not use any prior knowledge of recorded cells’ properties (e.g., their types or receptive fields), or any prior knowledge of the stimulus structure, to carry out the decoding; as a result, our decoding filters could, at least in principle, be used to decode any stimulus. A combination of sparse prior over decoding filter coefficients and a high-dimensional stimulus revealed a surprisingly local and stereotyped manner in which the retinal code could be read out. This is in stark contrast to previous work using simple stimuli where the readout was distributed and the resulting decoding filters had no general interpretation [12]. While our filters and consequently the “decoding fields” were recovered under a particular stimulus class and thus nominally depend on stimulus statistics, it is interesting to speculate whether the retina could adaptively change its encoding properties so as to keep the decoding representations constant, as has recently been suggested [12–14, 54]. Similarity between decoding and receptive fields and generalization to stimuli with different number of discs provide limited circumstantial support for this idea, but a definite answer can only emerge from dedicated experiments that specifically test the stability of decoders under rich stimuli with different statistical structure.

The performance of linear decoders was further improved by using nonlinear decoding. The improvement was significant, systematic, and reproducible: we observed it at nearly all sites, irrespectively of how many relevant cells we decoded from, when decoding from all recorded cells jointly or a mosaic of a single type, and also in a repeat experiment. Furthermore, a very different nonlinear regression method—a multi-layer neural network trained with standard deep learning tools—recapitulated quantitatively the results of kernelized decoding. The performance improvement of nonlinear methods is nontrivial, because the increased expressive power of nonlinear methods comes at a cost of potentially overfitting models to data; this was evident also in our failed first attempt to apply kernelized decoding to the whole recorded population, instead of only to the relevant cells selected by sparse linear decoder at every site. The performance improvement depended crucially on the spike-history dependence in individual spike trains but only slightly on cell-cell noise correlations. Previous work also explored the role of cell-cell noise correlations for decoding: while no impact of cell-cell noise correlations on decoding performance was found in mouse retinas exposed to white noise and natural scene stimulation [21], Pillow and colleagues report that the inclusion of cell-cell noise correlations in model-based decoding increased the stimulus information by about ∼20% [15]. We also observe a significant, 10 − 20% decrease in decoding performance if cell-cell noise correlations are removed from the test-set spike trains, with decoders trained on intact rasters. Our largest effect, however, comes from spike-history dependencies. Short-term history dependence in ganglion cells is mostly due to refractoriness, and including spike-history dependences of up to 40 ms after the spike did not substantially change the decoding performance from primate parasol cells [15]. In contrast, our spike-history dependencies extend over much longer times and modulate spiking structure over 100 ms or more, in temporal windows relevant for decoding; removing these dependencies drastically decreased the performance of nonlinear decoders. Consistent and robust results using two entirely different nonlinear regression methods, backed by simulations using GLM-model neurons, provide compelling evidence that spike-history dependencies indeed enable low-error stimulus reconstruction.

What are the methodological advances presented in our work? While sparse and nonlinear regression methods used here are standard methods in statistics, they have typically not been applied to spiking neural data for complex stimulus reconstruction. Nevertheless, we show here that they should provide a tractable way of studying how rich signals are represented in other parts of the brain without making explicit assumptions about the encoding process, thereby providing a complementary, decoder-centric alternative to Bayes inversion of probabilistic encoding models. Second, even though the inner workings of nonlinear methods are notoriously difficult to interpret intuitively, our analysis suggests that controlled manipulations of spike train statistics can provide valuable insights into which spike train features matter for decoding and which do not. Finally, we suggest the “pixel-by-pixel” decoding approach as an alternative way to shed light on the functional contributions of different cell types to stimulus representation. While beyond the scope of this paper, one could decode stimuli from individual mosaics of the same type, or from their combinations, and compare the decoding performance (and resulting errors) to that of a complete population.

What are the general implications of our results? The high-dimensional nature of our stimulus forced us to decode the movie “pixel-by-pixel,” rather than trying to decode its compact representation. This, in turn, focused our attention on the intermittent nature of signals to be decoded: at any given site, the luminance trace switched between epochs where nothing changed locally, and periods where the trace was fluctuating in time. Such intermittency is common to many natural stimuli across different sensory modalities [55, 56], and therefore must shape the way in which sensory information is encoded [57–59]. From the decoding perspective, it can, however, also pose a serious challenge: since neurons might be similarly active irrespective of whether the stimulus fluctuates locally or not, a downstream processing layer would have to suppress “hallucinations” in response to upstream network-driven or spontaneous activity (cf. [60]). This issue could be especially acute in the sensory periphery. The retina is an information bottleneck that conveys the information to the central brain in an essentially feed-forward fashion. Spontaneous activity [32–35] thus appears problematic, since there is no clear “extra” signal that could tell the downstream processing whether the input received from the retina is spontaneous or stimulus-driven; we thus looked for an intrinsic signature in the spike trains themselves. In contrast, cortex, with its recurrent / feedback architecture clearly supports the notion of cortical states that could provide additional information on how activity from higher sensory areas should be interpreted (e.g., is it a reverberation or current, stimulus-driven activity). Indeed, spontaneous and persistent spiking is widespread in the cortex [61–64] and has even been documented to statistically mimic the structure of stimulus-evoked activity [65].

Here we proposed a simple mechanism to discriminate spontaneous from stimulus-driven activity using history dependence of neural spiking: because neuronal encoding is nonlinear, the effect of spike-history dependence on neural firing substantially differs between epochs in which the neuron also experiences a strong stimulus drive and epochs in which it does not. In such situations, nonlinear methods can discriminate between a true stimulus fluctuation and spontaneous-like firing from statistical structure intrinsic to individual spike trains, even when the mean firing rate doesn’t change appreciably between different epochs. This mechanism is not specific to the retina, and may well apply in other systems that display both stimulus-evoked and spontaneous activity.

## Supporting information

S1 Fig Decodable information is represented locally. **Top.** Average (± SD) number of contributing cells (red) and all cells (black), as a function of distance of the cell’s receptive field center to the site where the luminance trace is being decoded. **Bottom.** Average (± SD) single cell decoding performance as a function of distance to the site. Cells’ responses contain no decodable information for sites that are > 200 *μm* distant from their receptive field centers. Both analyses are done for the 10-disc stimulus.
(TIF)

S2 Fig Cells are continuously active, but their responses only contain decodable information about local luminance fluctuations. The following analyses are carried out with a 1-disc stimulus. **A:** Average (± SD) single cell decoding performance as a function of distance of the cell’s receptive field center to the site where the luminance trace is being decoded. **B:** Firing rates of ON (N = 14) and OFF (N = 34) cells as a function of the distance to the single moving disc. Both types of cells exhibit basal firing rates > 10 Hz when the disc is far away from their receptive fields. OFF cells increase their firing rate when the dark disc is less than 200 *μm* away. ON cells decrease their firing in response to the dark disc and their firing rate peaks at the 200 *μm* mark, probably corresponding with the stimulation of their surround by the dark disc. **C**: Same as in **B** but now the basal firing rate (measured at 1000 *μm*) has been subtracted for each cell to emphasize the stereotyped dynamics of the cells’ activity. This analysis suggests that while cells are continuously active (even when the disc is far away and not stimulated by other discs, as in the case of S1 Fig), that activity does not contain decodable information about the luminance fluctuations farther than 200 *μm* from the receptive field center. In contrast, with simpler stimuli that stimulate retina more broadly (e.g., diffusively moving 1D bar), retinal ganglion cells encoded for the bar position in a distributed manner such that the stimulus could be decoded from multiple subsets of cells and even from cells whose receptive field centers were very distant from the bar position [12].
(TIF)

S3 Fig Examples of decoding fields for 6 different cells. Each pixel corresponds to a site (of a 50 × 50 grid) and the color code represents the decoding filter of the cell at that particular site and time. The filters have been normalized such that the site of maximum variation has variance equal to 1. The white noise receptive field center of each cell is shown for reference (black ellipse).
(TIF)

S4 Fig Decoding filters of best contributing cells have a stereotyped shape. Decoding filters of the 1st and 2nd best contributing cells across sites, normalized to unit variance. The shape of the filters is very similar and differs primarily by a multiplicative scaling factor. We could assume a universal temporal profile for all cells at all sites, and perform the decoding by fitting a single multiplicative scale parameter (with a sign, to account for ON/OFF differences) per cell per site, with less than 6% drop in FVE on the 10-disc stimulus, compared to the model in the main text that makes no assumption about stereotyped filter shapes.
(TIF)

S5 Fig Decoding preferentially recruits OFF cells. Bias in the ON/OFF cells ratio plotted separately for the single-, two- and three-best-cell decoding subsets for each site. By looking in detail at the contribution of ON vs OFF cells to stimulus reconstruction at every site we find a clear bias for OFF cells relative to the prediction based on random draws from the local ON/OFF composition (see Methods). This OFF bias matched our expectation for optimally tracking dark discs displayed in our experiments.
(TIF)

S6 Fig Redundancy of decodable information about local luminance traces. Average fractional decrease in linear decoding performance across sites when progressively removing cells (± SD). At each site cells are removed in order of importance, according to their decoding filter norm. We compare the performance when decoding with all available cells (FVE(all)) and when decoding without the first *N* contributing cells (FVE). This is one way to estimate the redundancy in the population response. Removing 4-5 cells halves decoding performance, suggesting that the necessary information for linear decoding is contained in a small number of cells. This is in contrast with previous work [12], where we found that the information about the position of a moving bar was encoded in a highly redundant manner. In that work we were able to construct 5 disjoint subsets of cells (from 2 to 10 cells in size) from which the position of the bar could be decoded with low error. Together with S2 Fig this suggests that complex stimuli used here lead to much more local and less redundant responses that carry stimulus information (compared to e.g., diffusive bar motion), even though the retina is broadly active in both cases.
(TIF)

S7 Fig Choice of best subset of cells for kernelized nonlinear decoding. Decoding error of the nonlinear decoder is plotted as a function of the number of cells considered for six different sites. Cells are ordered by the decreasing L1 norm of their linear filters (i.e., cell 1 is the best contributing cell, etc). The optimal subset (circle) is chosen through cross validation to minimize the error on the training set. The error of the nonlinear decoder on the test set is shown for comparison.
(TIF)

S8 Fig Examples of decoded movie frames with linear and kernelized nonlinear decoding. Black contour marks the region of good cell coverage where linear decoding performs at *FVE* > 0.4; green circles in decoded frames correspond to true positions of the discs.
(TIF)

S9 Fig Decoding from single cell type mosaics. **A**: OFF-cell mosaic (N = 33). In the left-most panel temporal receptive field and spatial receptive field centers are shown. Center panel shows the performance of the linear decoders in space (measured as FVE). The contour lines mark the boundary FVE = 0.3, and we only consider sites within this boundary to compute the average decoder performance (± SD across sites), achievable using increasing numbers of cells with highest L1 filter norm (right-most panel). For nonlinear decoding, “All” is the optimal subset that maximizes performance. **B**: ON-cell mosaic (N = 22). Details equivalent to **A**. In both cases, nonlinear decoding substantially improves on linear.
(TIF)

S10 Fig Decoding performance for a repeat experiment with a retina of a different rat. Average decoder performance (± SD across sites), achievable using increasing number of cells with highest L1 filter norm. For kernelized nonlinear decoding, “All” is the optimal subset that maximizes performance. In the repeat experiment we isolated 64 retinal ganglion cells and identified 125 sites where linear decoding performed at FVE>0.4.
(TIF)

S11 Fig Kernelized nonlinear classifiers outperform linear classifiers on multiple cells and single cell responses. **A:** Performance (F-score) of linear and nonlinear classifiers for different sites (black dots). Inset: average (± SEM) over sites is significantly different (p<0.001). **B:** Performance (F-score) of linear and nonlinear classifiers for each site when trained and tested from a single cell response (the best cell for each site). Average performance is shown in the inset (± SEM) and the differences between linear and nonlinear are significant (p<0.001).
(TIF)

S12 Fig Kernelized nonlinear decoders predict more constant signal under blackout stimulation. **A**: Variance of the single cell decoded traces from spontaneous activity (average across sites ± SEM). The decoders are trained on 10-discs stimulus and tested on the responses recorded during blackout condition (full darkness). Nonlinear decoders produce traces with significantly lower variance (p<0.001). **B**: Example of mean-subtracted blackout decoded traces from a single cell spike train (bottom) with linear and nonlinear decoders.
(TIF)

S13 Fig Kernelized nonlinear classifiers rely on spike-history dependencies. Decrease in classifier performance (F-score) when spike-history dependencies or noise correlations are removed (average ± SEM across sites); percentages report fractional differences relative to the original performance.
(TIF)

S14 Fig Kernelized nonlinear decoders (classifiers) rely on spike-history dependences when decoding (classifying) single cell responses. Changes in single cell decoders and classifiers performance when spike-history dependencies are removed. We show differences in average decoding error (MSE) for the decoder and differences in performance (F-score) for the classifier (± SEM). The percentages shown stand for average fractional difference with respect to the original performance (before removing correlations). The differences are statistically significant in both cases (p<0.001).
(TIF)

S15 Fig Spike count variance-to-mean ratio *F* is similar for responses at locally constant luminance and spontaneous activity, and differs for locally fluctuating luminance. Variance-to-mean ratio *F*, under different stimulus conditions, of the spike count distributions P(K) of the best cell for each site (average over sites ± SD). “Spontaneous” is the activity under blackout condition (no stimulus). Values of *F* of “spontaneous” and “constant” activities are not significantly different, pointing at similarities between these two responses. On the contrary, both of them are clearly different from the activity under fluctuating stimulation (p<0.001).
(TIF)

S16 Fig Interspike interval distributions differ at locally fluctuating luminance and locally constant luminance or spontaneous activity. Logarithmic differences between the Inter-Spike-Interval (ISI) distributions under fluctuating [*p*_*f*_(*ISI*)] and constant [*p*_*c*_(*ISI*)] stimulus and between fluctuating and spontaneous activity [*p*_*s*_(*ISI*)]. The distributions are computed for the single best cell at each site. The average across sites (± SD) is shown. Similarly to the spike count distributions *P*(*K*), the *ISI* distributions show activity under constant stimulation to be more regular and dominated by *ISI* between 75 ms and 175 ms. *ISI* outside this range are more common during fluctuating stimulation.
(TIF)

S17 Fig GLM models account well for the firing rates of cells recorded in the 10-disc experiment. Three examples of GLM fits of real cells in our data set. On the left we show the fitted filters, nonlinearity, and spike history term that compose the model. On the right we show real and model generated repeated stimulus raster responses, and compare the real and predicted PSTH.
(TIFF)

S18 Fig Classifier performance for constant vs fluctuating local luminance peaks at an intermediate value of spike-history dependencies. Average classifier performance (F-score) as a function of *α* (see Fig 4 in the main text). The error bars correspond to standard deviation over 10 different realizations of the spike trains generated from the model for each value of *α*.
(TIF)

S19 Fig Decoding error in constant and fluctuating segments is optimal for intermediate value of spike-history dependencies. (TIF)

S20 Fig Deep neural network architecture and its protoype outputs. **A**: The used artifical neural network is a fully connected feed forward network with three hidden layers, each 150 units and hyperbolic tangent activation function. The networks learns to map the response, given by the windowed spike train (Input), to the stimulus (Output). **B**: For each unit in the last hidden layer we mark its corresponding output activation visualiuzed by contour lines. The white background indicates the selected cells as in Fig 1F. Note that due to sparsity regularization (prefering networks with smaller number of weights) only 41 cells have non-zero connections to the output in the presented instance.
(TIF)

S21 Fig Neural network decoding fields. Examples of decoding fields for the same 6 cells of S3 Fig. The white noise receptive field center of each cell is shown for reference (blue ellipse). It is obtained by activating the network with a single spike of the cell at the specified time. The output is normalized as in S3 Fig.
(TIF)

S1 Video Segment of real and nonlinearly decoded movie. The black contour marks the region of good cell coverage where linear decoding performs at *FVE* > 0.4. Green contours of the discs in the real stimulus have been superimposed on the decoded movie as a visual aid for comparison purposes.
(MP4)
