# Supplementary figures and images for: Nonlinear decoding of a complex movie from the mammalian retina
Source: PLoS Comput Biol. 2018 May 10;14(5):e1006057. doi: 10.1371/journal.pcbi.1006057 (PMC5944913; doi:10.1371/journal.pcbi.1006057)

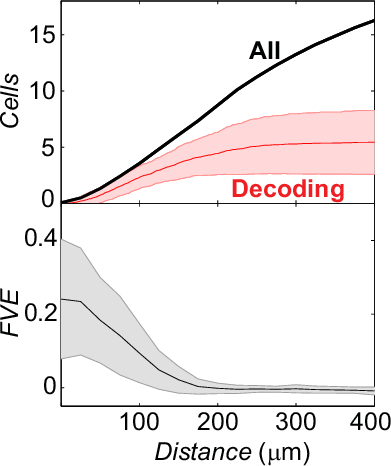

Supplement: S1 Fig — Top. Average (± SD) number of contributing cells (red) and all cells (black), as a function of distance of the cell’s receptive field center to the site where the luminance trace is being decoded. Bottom. Average (± SD) single cell decoding performance as a function of distance to the site. Cells’ responses contain no decodable information for sites that are > 200 μm distant from their receptive field centers. Both analyses are done for the 10-disc stimulus. (TIF) [file pcbi.1006057.s001.tif]

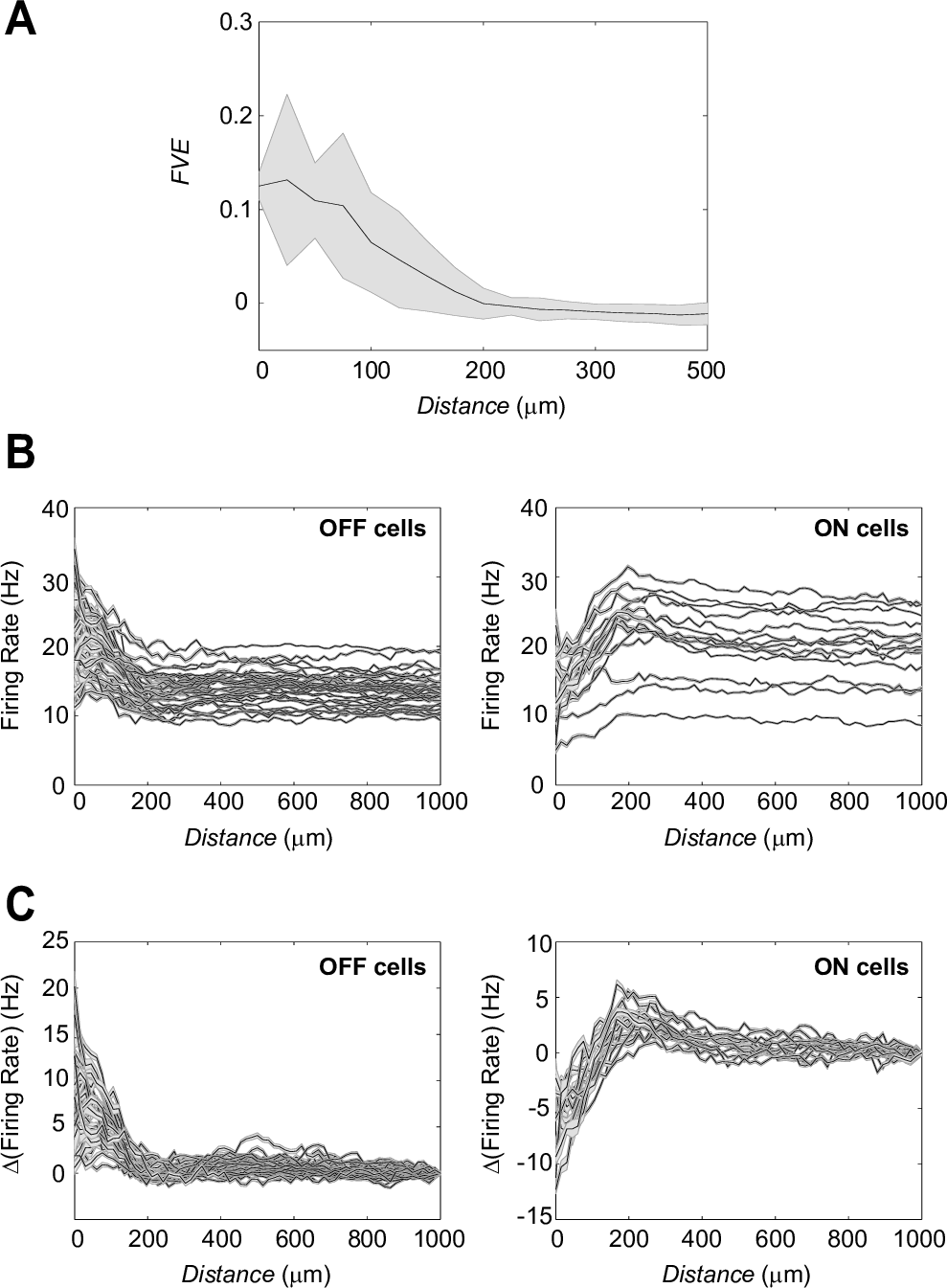

Supplement: S2 Fig — The following analyses are carried out with a 1-disc stimulus. A: Average (± SD) single cell decoding performance as a function of distance of the cell’s receptive field center to the site where the luminance trace is being decoded. B: Firing rates of ON (N = 14) and OFF (N = 34) cells as a function of the distance to the single moving disc. Both types of cells exhibit basal firing rates > 10 Hz when the disc is far away from their receptive fields. OFF cells increase their firing rate when the dark disc is less than 200 μm away. ON cells decrease their firing in response to the dark disc and their firing rate peaks at the 200 μm mark, probably corresponding with the stimulation of their surround by the dark disc. C: Same as in B but now the basal firing rate (measured at 1000 μm) has been subtracted for each cell to emphasize the stereotyped dynamics of the cells’ activity. This analysis suggests that while cells are continuously active (even when the disc is far away and not stimulated by other discs, as in the case of S1 Fig), that activity does not contain decodable information about the luminance fluctuations farther than 200 μm from the receptive field center. In contrast, with simpler stimuli that stimulate retina more broadly (e.g., diffusively moving 1D bar), retinal ganglion cells encoded for the bar position in a distributed manner such that the stimulus could be decoded from multiple subsets of cells and even from cells whose receptive field centers were very distant from the bar position [12]. (TIF) [file pcbi.1006057.s002.tif]

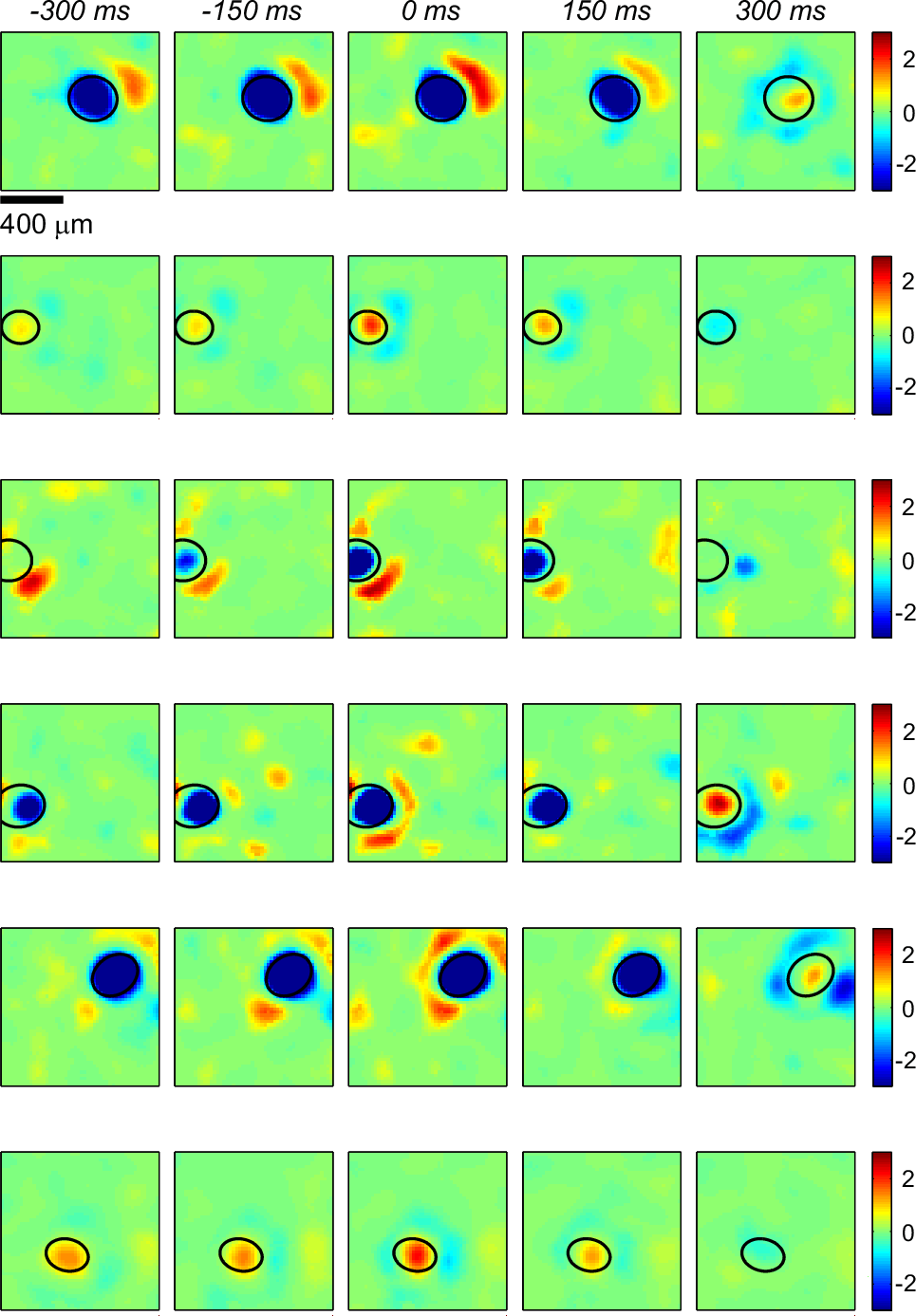

Supplement: S3 Fig — Each pixel corresponds to a site (of a 50 × 50 grid) and the color code represents the decoding filter of the cell at that particular site and time. The filters have been normalized such that the site of maximum variation has variance equal to 1. The white noise receptive field center of each cell is shown for reference (black ellipse). (TIF) [file pcbi.1006057.s003.tif]

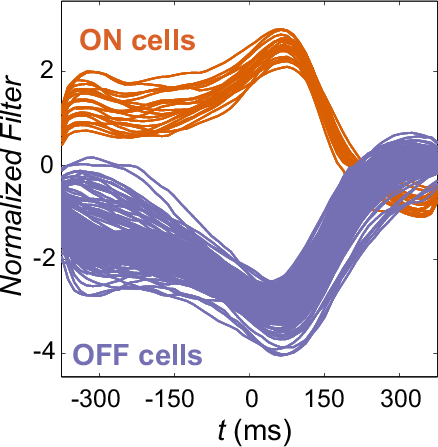

Supplement: S4 Fig — Decoding filters of the 1st and 2nd best contributing cells across sites, normalized to unit variance. The shape of the filters is very similar and differs primarily by a multiplicative scaling factor. We could assume a universal temporal profile for all cells at all sites, and perform the decoding by fitting a single multiplicative scale parameter (with a sign, to account for ON/OFF differences) per cell per site, with less than 6% drop in FVE on the 10-disc stimulus, compared to the model in the main text that makes no assumption about stereotyped filter shapes. (TIF) [file pcbi.1006057.s004.tif]

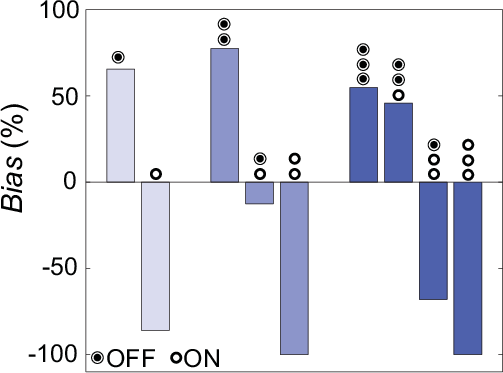

Supplement: S5 Fig — Bias in the ON/OFF cells ratio plotted separately for the single-, two- and three-best-cell decoding subsets for each site. By looking in detail at the contribution of ON vs OFF cells to stimulus reconstruction at every site we find a clear bias for OFF cells relative to the prediction based on random draws from the local ON/OFF composition (see Methods). This OFF bias matched our expectation for optimally tracking dark discs displayed in our experiments. (TIF) [file pcbi.1006057.s005.tif]

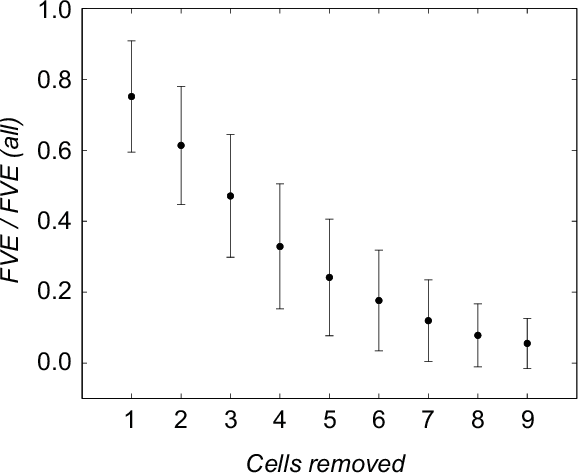

Supplement: S6 Fig — Average fractional decrease in linear decoding performance across sites when progressively removing cells (± SD). At each site cells are removed in order of importance, according to their decoding filter norm. We compare the performance when decoding with all available cells (FVE(all)) and when decoding without the first N contributing cells (FVE). This is one way to estimate the redundancy in the population response. Removing 4-5 cells halves decoding performance, suggesting that the necessary information for linear decoding is contained in a small number of cells. This is in contrast with previous work [12], where we found that the information about the position of a moving bar was encoded in a highly redundant manner. In that work we were able to construct 5 disjoint subsets of cells (from 2 to 10 cells in size) from which the position of the bar could be decoded with low error. Together with S2 Fig this suggests that complex stimuli used here lead to much more local and less redundant responses that carry stimulus information (compared to e.g., diffusive bar motion), even though the retina is broadly active in both cases. (TIF) [file pcbi.1006057.s006.tif]

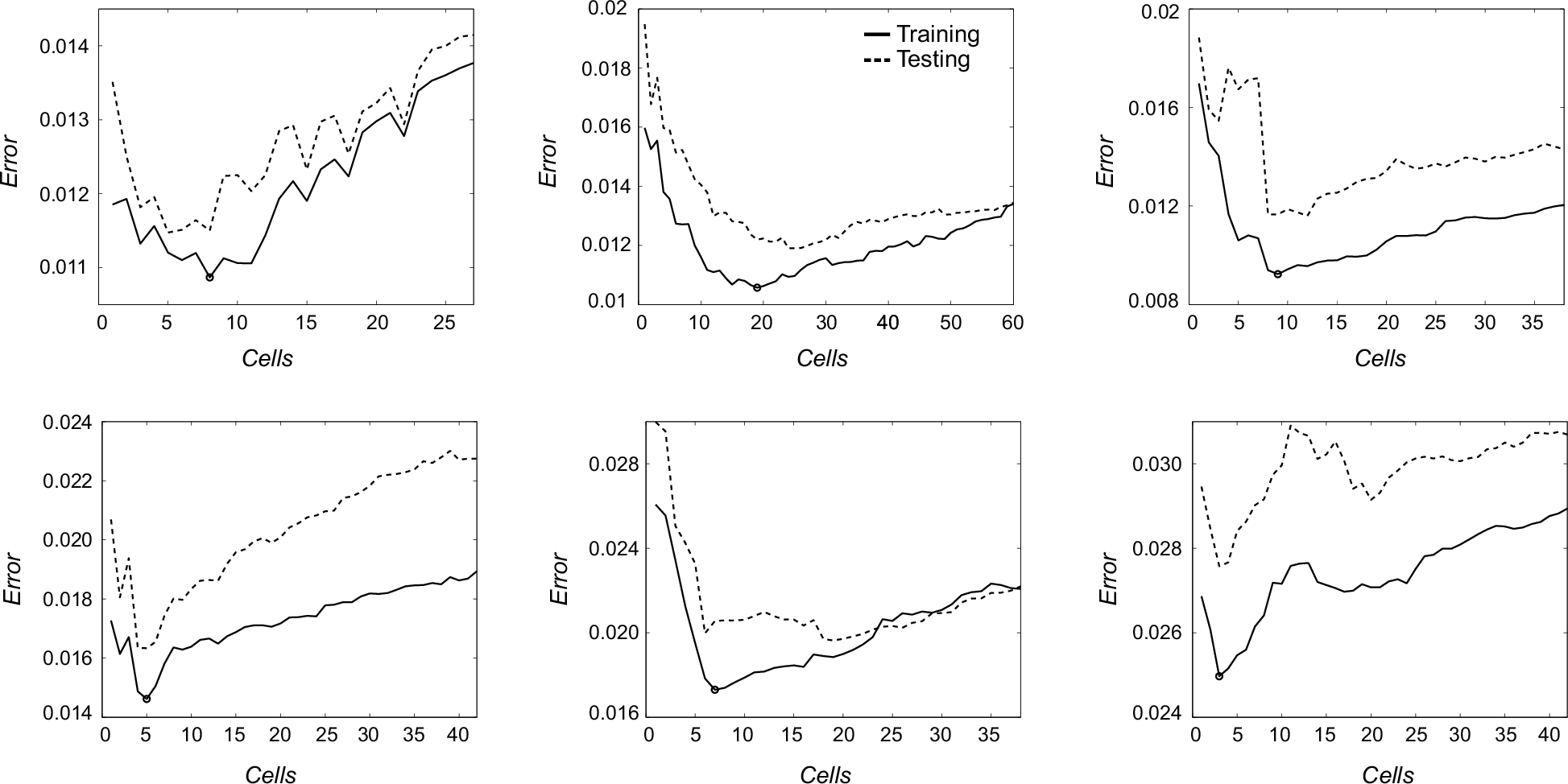

Supplement: S7 Fig — Decoding error of the nonlinear decoder is plotted as a function of the number of cells considered for six different sites. Cells are ordered by the decreasing L1 norm of their linear filters (i.e., cell 1 is the best contributing cell, etc). The optimal subset (circle) is chosen through cross validation to minimize the error on the training set. The error of the nonlinear decoder on the test set is shown for comparison. (TIF) [file pcbi.1006057.s007.tif]

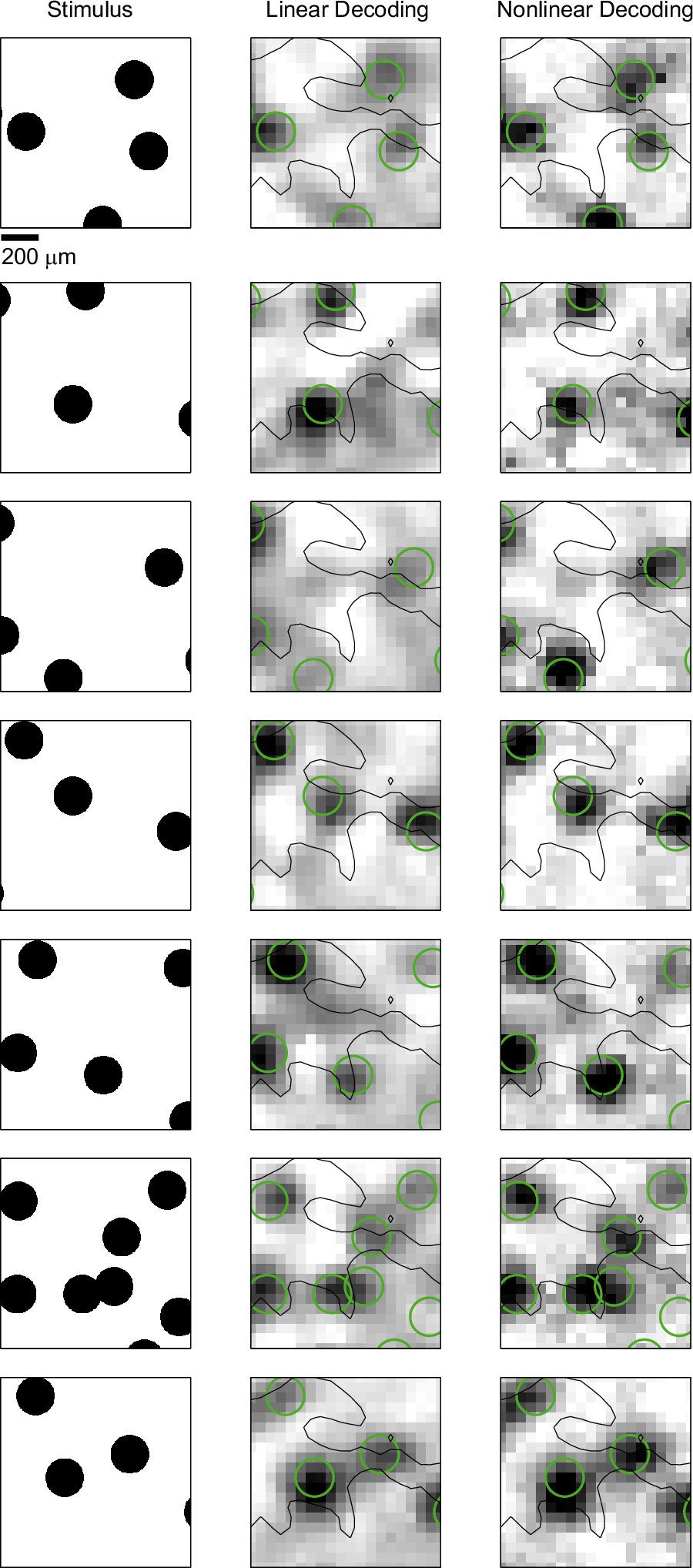

Supplement: S8 Fig — Black contour marks the region of good cell coverage where linear decoding performs at FVE > 0.4; green circles in decoded frames correspond to true positions of the discs. (TIF) [file pcbi.1006057.s008.tif]

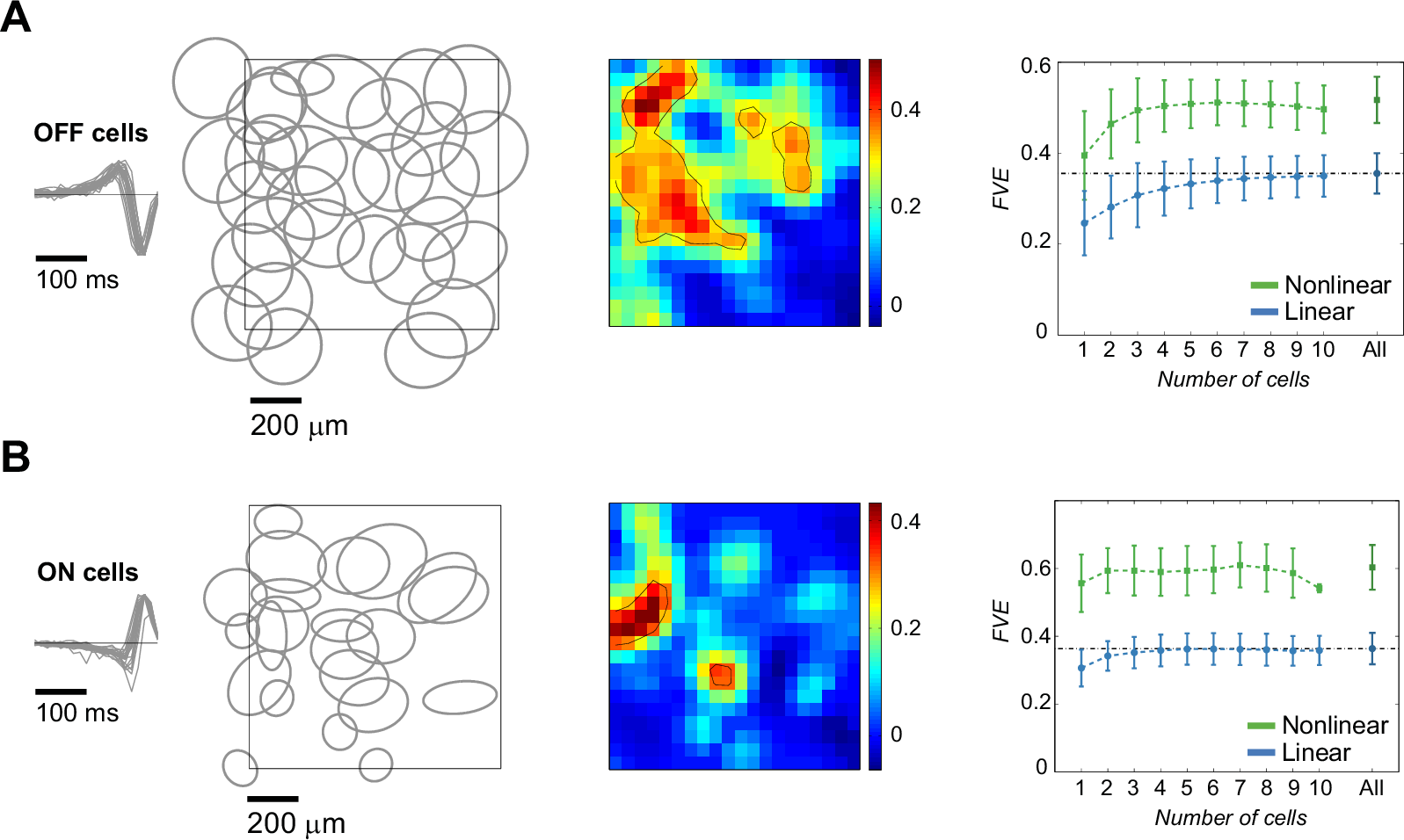

Supplement: S9 Fig — A: OFF-cell mosaic (N = 33). In the left-most panel temporal receptive field and spatial receptive field centers are shown. Center panel shows the performance of the linear decoders in space (measured as FVE). The contour lines mark the boundary FVE = 0.3, and we only consider sites within this boundary to compute the average decoder performance (± SD across sites), achievable using increasing numbers of cells with highest L1 filter norm (right-most panel). For nonlinear decoding, “All” is the optimal subset that maximizes performance. B: ON-cell mosaic (N = 22). Details equivalent to A. In both cases, nonlinear decoding substantially improves on linear. (TIF) [file pcbi.1006057.s009.tif]

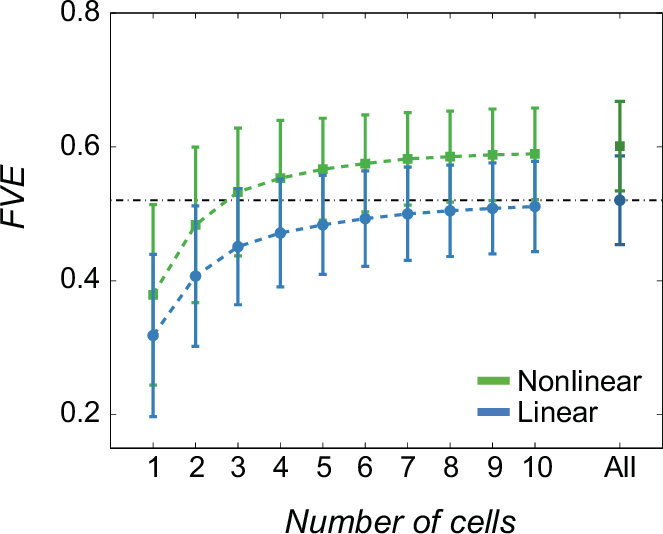

Supplement: S10 Fig — Average decoder performance (± SD across sites), achievable using increasing number of cells with highest L1 filter norm. For kernelized nonlinear decoding, “All” is the optimal subset that maximizes performance. In the repeat experiment we isolated 64 retinal ganglion cells and identified 125 sites where linear decoding performed at FVE>0.4. (TIF) [file pcbi.1006057.s010.tif]

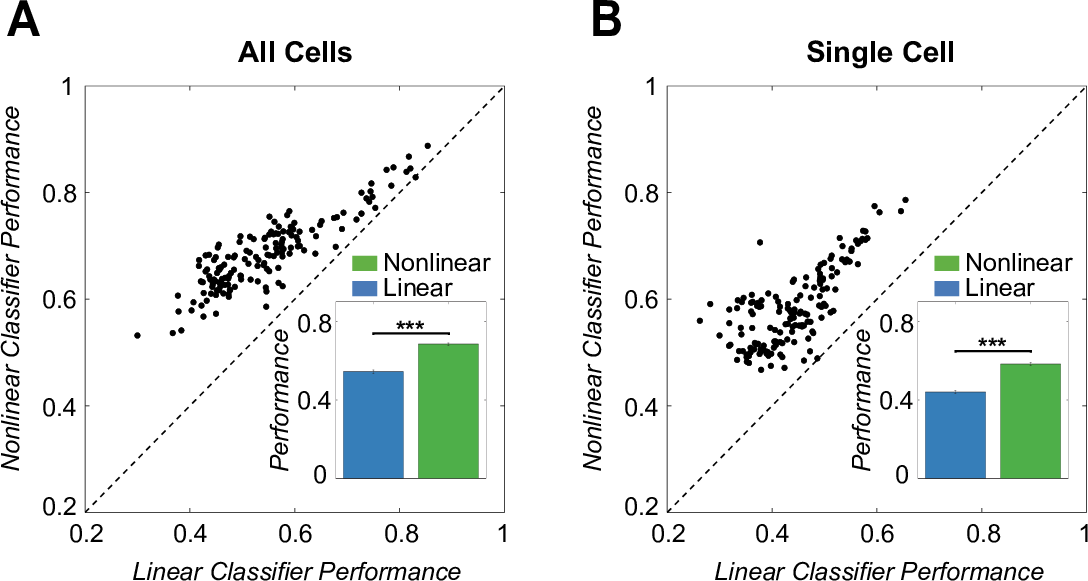

Supplement: S11 Fig — A: Performance (F-score) of linear and nonlinear classifiers for different sites (black dots). Inset: average (± SEM) over sites is significantly different (p<0.001). B: Performance (F-score) of linear and nonlinear classifiers for each site when trained and tested from a single cell response (the best cell for each site). Average performance is shown in the inset (± SEM) and the differences between linear and nonlinear are significant (p<0.001). (TIF) [file pcbi.1006057.s011.tif]

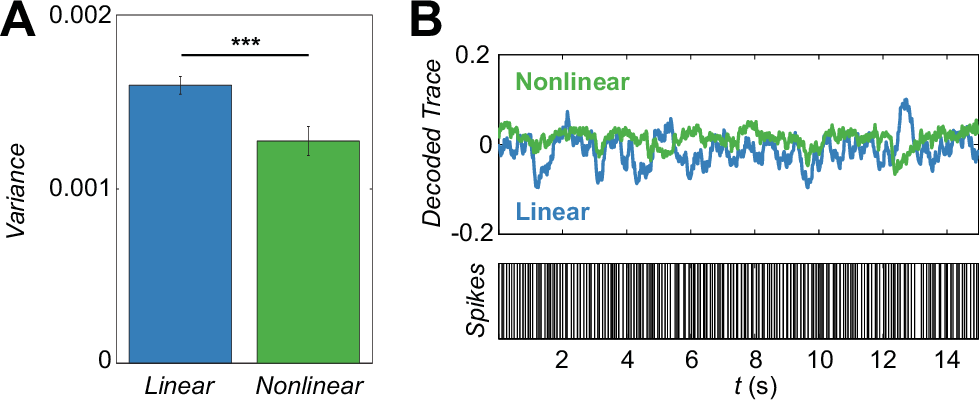

Supplement: S12 Fig — A: Variance of the single cell decoded traces from spontaneous activity (average across sites ± SEM). The decoders are trained on 10-discs stimulus and tested on the responses recorded during blackout condition (full darkness). Nonlinear decoders produce traces with significantly lower variance (p<0.001). B: Example of mean-subtracted blackout decoded traces from a single cell spike train (bottom) with linear and nonlinear decoders. (TIF) [file pcbi.1006057.s012.tif]

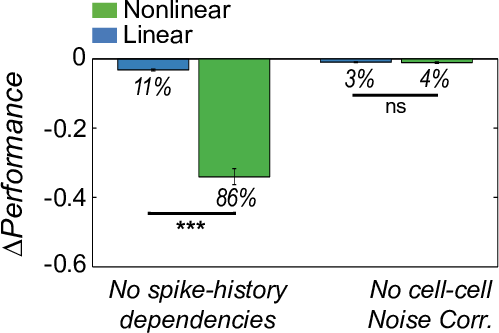

Supplement: S13 Fig — Decrease in classifier performance (F-score) when spike-history dependencies or noise correlations are removed (average ± SEM across sites); percentages report fractional differences relative to the original performance. (TIF) [file pcbi.1006057.s013.tif]

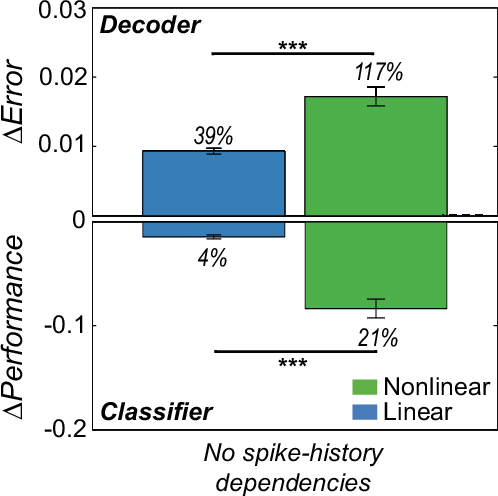

Supplement: S14 Fig — Changes in single cell decoders and classifiers performance when spike-history dependencies are removed. We show differences in average decoding error (MSE) for the decoder and differences in performance (F-score) for the classifier (± SEM). The percentages shown stand for average fractional difference with respect to the original performance (before removing correlations). The differences are statistically significant in both cases (p<0.001). (TIF) [file pcbi.1006057.s014.tif]

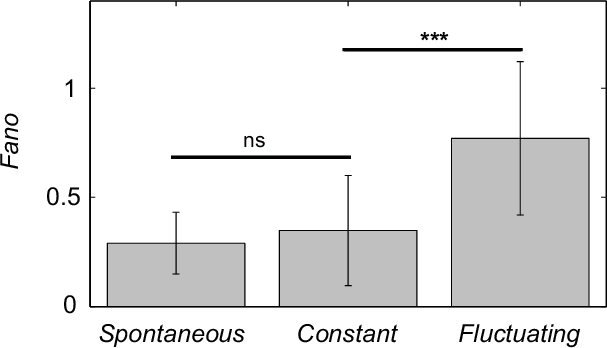

Supplement: S15 Fig — Variance-to-mean ratio F, under different stimulus conditions, of the spike count distributions P(K) of the best cell for each site (average over sites ± SD). “Spontaneous” is the activity under blackout condition (no stimulus). Values of F of “spontaneous” and “constant” activities are not significantly different, pointing at similarities between these two responses. On the contrary, both of them are clearly different from the activity under fluctuating stimulation (p<0.001). (TIF) [file pcbi.1006057.s015.tif]

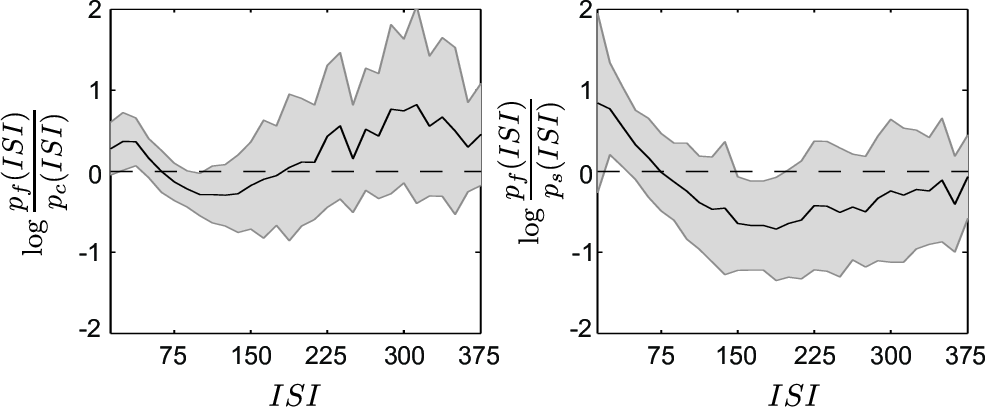

Supplement: S16 Fig — Logarithmic differences between the Inter-Spike-Interval (ISI) distributions under fluctuating [pf(ISI)] and constant [pc(ISI)] stimulus and between fluctuating and spontaneous activity [ps(ISI)]. The distributions are computed for the single best cell at each site. The average across sites (± SD) is shown. Similarly to the spike count distributions P(K), the ISI distributions show activity under constant stimulation to be more regular and dominated by ISI between 75 ms and 175 ms. ISI outside this range are more common during fluctuating stimulation. (TIF) [file pcbi.1006057.s016.tif]

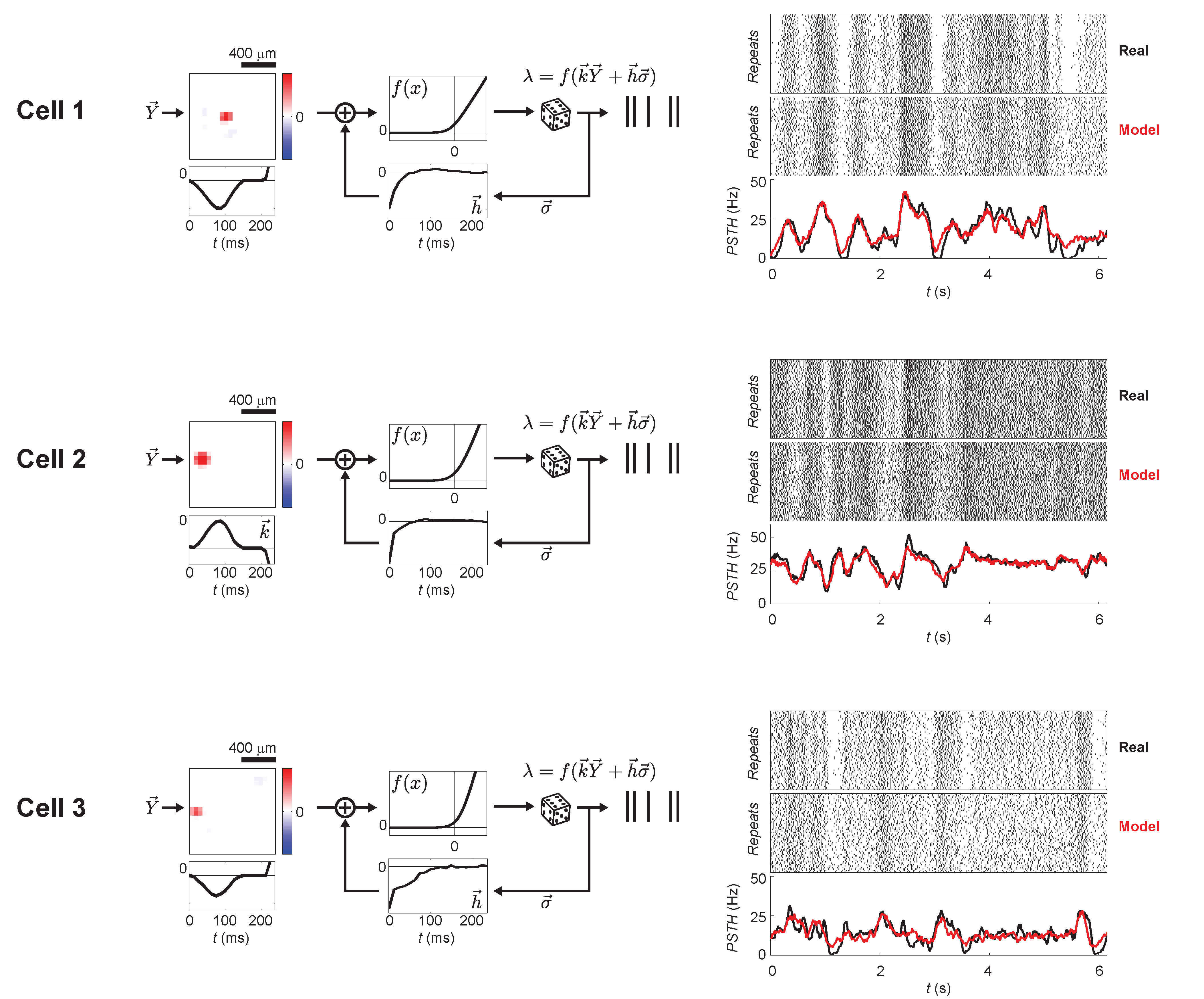

Supplement: S17 Fig — Three examples of GLM fits of real cells in our data set. On the left we show the fitted filters, nonlinearity, and spike history term that compose the model. On the right we show real and model generated repeated stimulus raster responses, and compare the real and predicted PSTH. (TIFF) [file pcbi.1006057.s017.tiff]

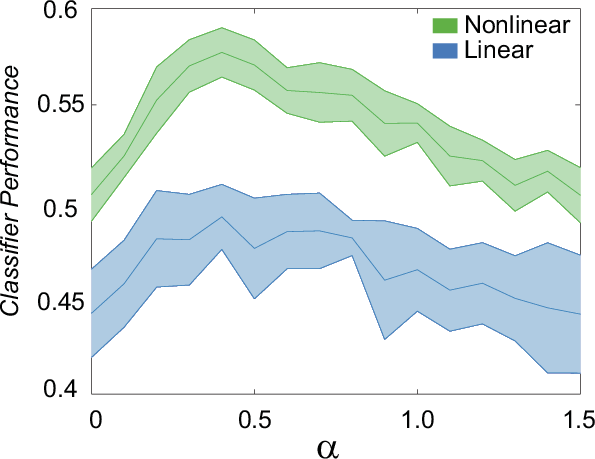

Supplement: S18 Fig — Average classifier performance (F-score) as a function of α (see Fig 4 in the main text). The error bars correspond to standard deviation over 10 different realizations of the spike trains generated from the model for each value of α. (TIF) [file pcbi.1006057.s018.tif]

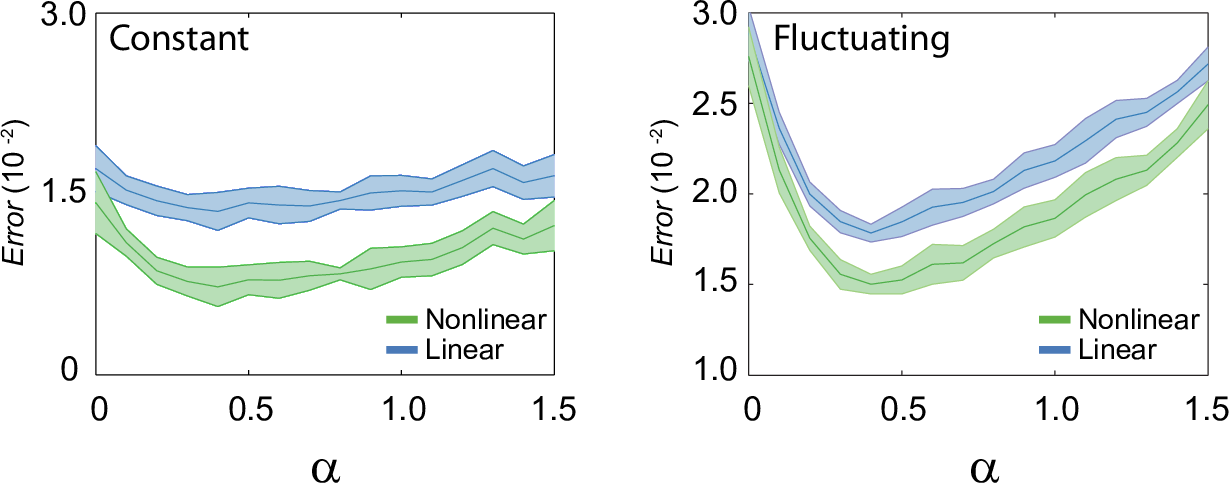

Supplement: S19 Fig — (TIF) [file pcbi.1006057.s019.tif]

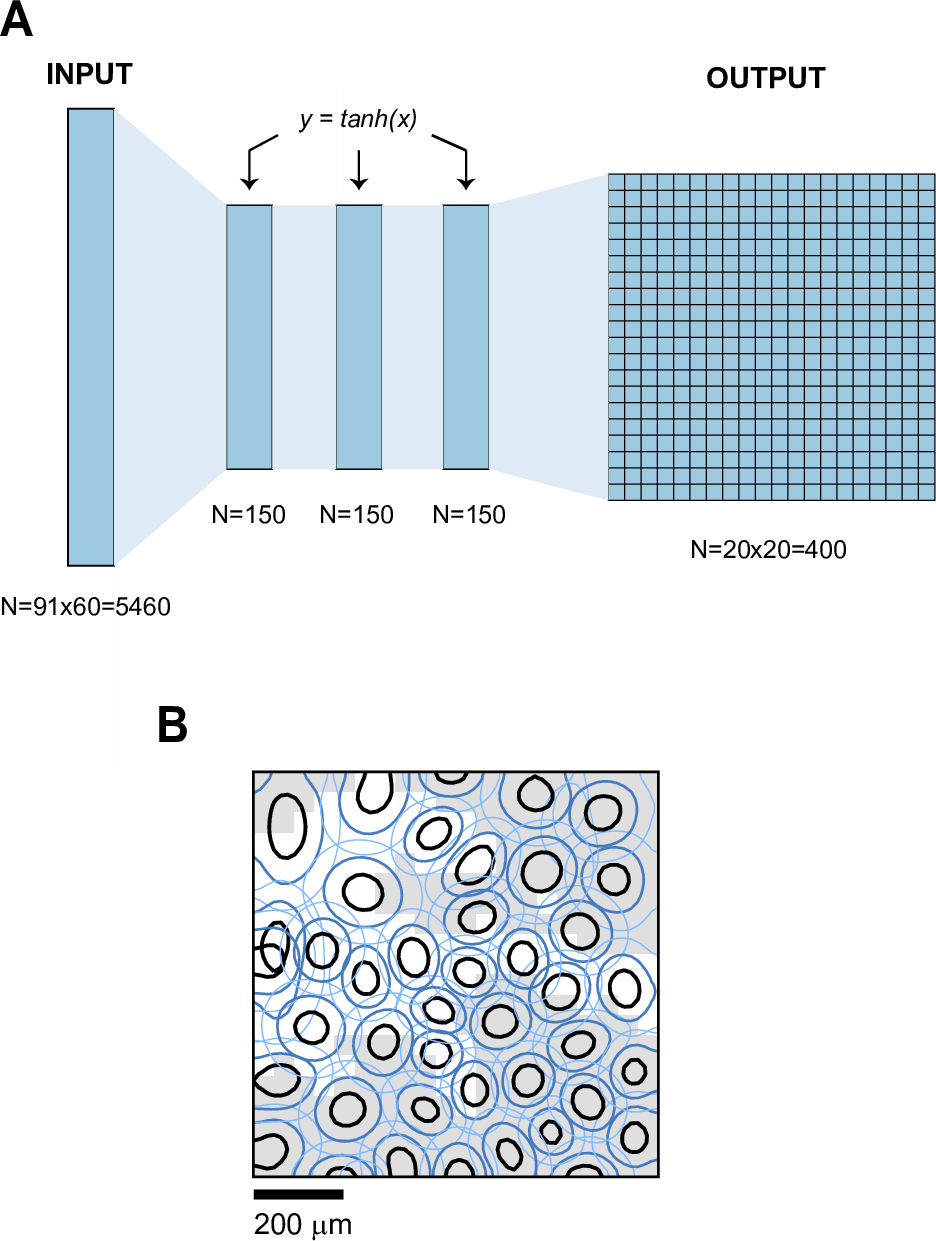

Supplement: S20 Fig — A: The used artifical neural network is a fully connected feed forward network with three hidden layers, each 150 units and hyperbolic tangent activation function. The networks learns to map the response, given by the windowed spike train (Input), to the stimulus (Output). B: For each unit in the last hidden layer we mark its corresponding output activation visualiuzed by contour lines. The white background indicates the selected cells as in Fig 1F. Note that due to sparsity regularization (prefering networks with smaller number of weights) only 41 cells have non-zero connections to the output in the presented instance. (TIF) [file pcbi.1006057.s020.tif]

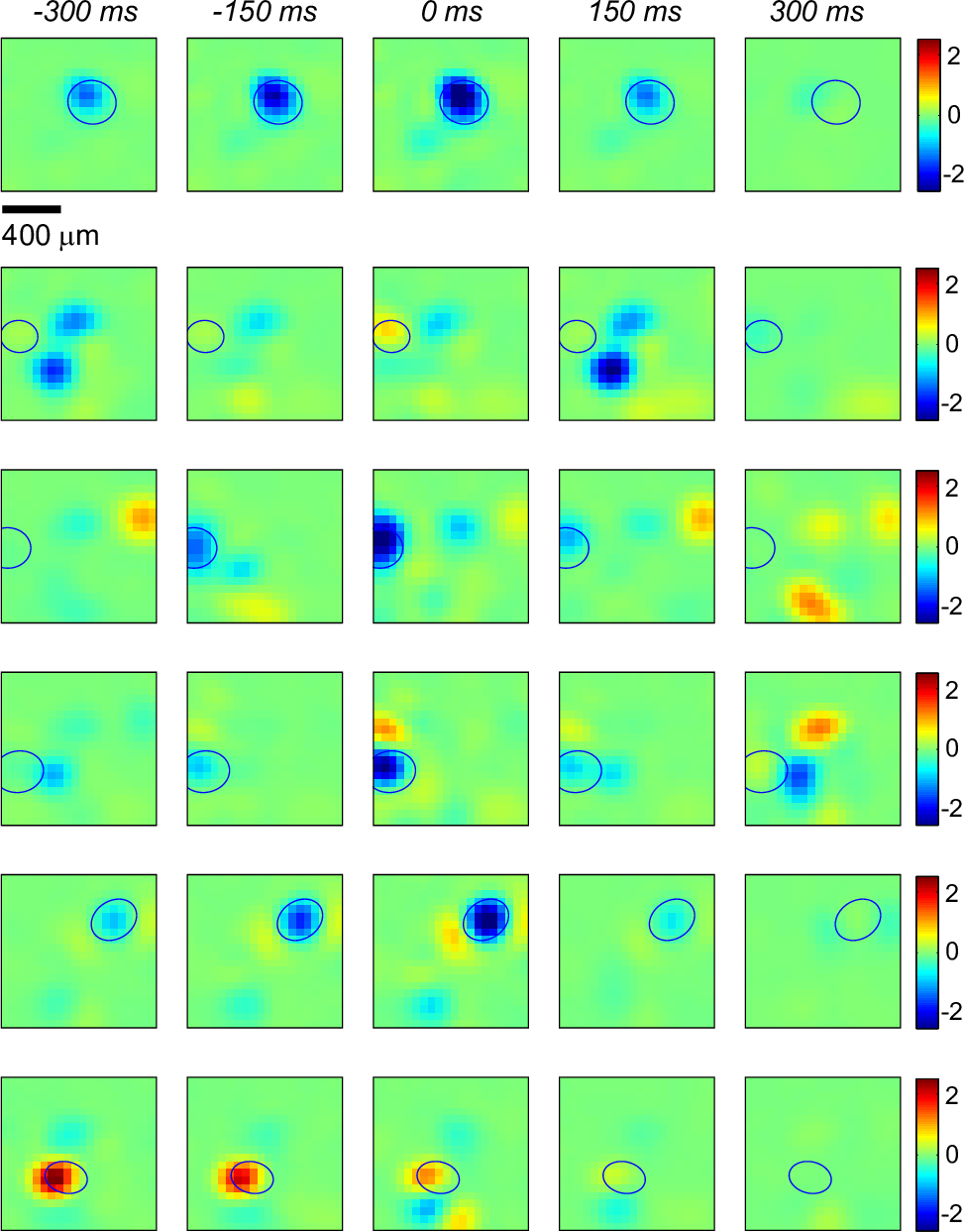

Supplement: S21 Fig — Examples of decoding fields for the same 6 cells of S3 Fig. The white noise receptive field center of each cell is shown for reference (blue ellipse). It is obtained by activating the network with a single spike of the cell at the specified time. The output is normalized as in S3 Fig. (TIF) [file pcbi.1006057.s021.tif]
